# The Evolution of Oxytocin and Vasotocin Receptor Genes in Jawed Vertebrates: A Clear Case for Gene Duplications Through Ancestral Whole-Genome Duplications

**DOI:** 10.3389/fendo.2021.792644

**Published:** 2022-02-03

**Authors:** Daniel Ocampo Daza, Christina A. Bergqvist, Dan Larhammar

**Affiliations:** ^1^Subdepartment of Evolution and Development, Department of Organismal Biology, Uppsala University, Uppsala, Sweden; ^2^Department of Molecular and Cell Biology, University of California Merced, Merced, CA, United States; ^3^Department of Neuroscience, Science for Life Laboratory, Uppsala University, Uppsala, Sweden

**Keywords:** GPCR, GPCR evolution, oxytocin receptor, vasopressin receptor, isotocin receptor, vasotocin receptor, whole-genome duplication (WGD)

## Abstract

The neuronal and neuroendocrine peptides oxytocin (OT) and vasotocin (VT), including vasopressins, have six cognate receptors encoded by six receptor subtype genes in jawed vertebrates. The peptides elicit a broad range of responses that are specifically mediated by the receptor subtypes including neuronal functions regulating behavior and hormonal actions on reproduction and water/electrolyte balance. Previously, we have demonstrated that these six receptor subtype genes, which we designated *VTR1A*, *VTR1B*, *OTR*, *VTR2A*, *VTR2B* and *VTR2C*, arose from a syntenic ancestral gene pair, one VTR1/OTR ancestor and one *VTR2* ancestor, through the early vertebrate whole-genome duplications (WGD) called 1R and 2R. This was supported by both phylogenetic and chromosomal conserved synteny data. More recently, other studies have focused on confounding factors, such as the OTR/VTR orthologs in cyclostomes, to question this scenario for the origin of the OTR/VTR gene family; proposing instead less parsimonious interpretations involving only one WGD followed by complex series of chromosomal or segmental duplications. Here, we have updated the phylogeny of the OTR/VTR gene family, including a larger number of vertebrate species, and revisited seven representative neighboring gene families from our previous conserved synteny analyses, adding chromosomal information from newer high-coverage genome assemblies from species that occupy key phylogenetic positions: the polypteriform fish reedfish (*Erpetoichthys calabaricus*), the cartilaginous fish thorny skate (*Amblyraja radiata*) and a more recent high-quality assembly of the Western clawed frog (*Xenopus tropicalis*) genome. Our analyses once again add strong support for four-fold symmetry, i.e., chromosome quadruplication in the same time window as the WGD events early in vertebrate evolution, prior to the jawed vertebrate radiation. Thus, the evolution of the OTR/VTR gene family can be most parsimoniously explained by two WGD events giving rise to the six ancestral genes, followed by differential gene losses of VTR2 genes in different lineages. We also argue for more coherence and clarity in the nomenclature of OT/VT receptors, based on the most parsimonious scenario.

## 1 Introduction

The two mammalian neuronal and neuroendocrine peptides oxytocin and vasopressin are involved in numerous functions, some of the most notable of which are reproduction and social behavior for oxytocin and water/electrolyte balance and social behavior for vasopressin (1). Their chemical characterization was awarded the Nobel Prize in physiology or medicine to Vincent Du Vigneaud in 1955. As related peptides began to be isolated and characterized in the 1950s, including complete sequencing in several species, a tradition arose to give each new peptide with a unique sequence a distinct name (2). This resulted in a plethora of names, the most commonly used of which are isotocin (ray-finned fishes), mesotocin (non-eutherian tetrapods) and vasotocin (non-mammalian vertebrates). The most widespread vasopressin sequence in mammals was named arginine-vasopressin when it was discovered that the porcine sequence had replaced the arginine (Arg) residue on position 8 with lysine (Lys), thus lysine-vasopressin or lyspressin. This resulted in the abbreviation AVP for arginine-vasopressin. This can be misleading because the single-letter abbreviation for arginine is not A but R. This naming mania arose long before it was possible to assign orthology between evolutionary lineages with certainty, and before the importance of orthology and paralogy for gene naming was realized.

Today, we know that a single common ancestral pre-pro-peptide gene existed before the origin of vertebrates (3). The single gene in the vertebrate ancestor underwent a tandem duplication in the common ancestor of the jawed vertebrates (Gnathostomata). Rearrangements have subsequently happened in a few lineages such as placental mammals which have the two peptide genes in a tail-to-tail configuration (3). Nevertheless, it is clear that the peptides named oxytocin, isotocin and mesotocin are orthologous, as are vasopressins and vasotocin. Herein we will use the names oxytocin (OT) and vasotocin (VT) as recommended in the recent article by Theofanopoulou et al. (4). A recent peptide and receptor evolution review described invertebrate homologs (orthologs) of oxytocin and its receptors (5).

In mammals, the two peptides have four cognate receptor subtypes encoded by four distinct genes: *AVPR1A*, *AVPR1B*, *AVPR2* and *OXTR*. The actions of OT are mediated by the OT receptor (OTR) and include both behavioral and gonadal regulation and, in mammals, the powerful contractions of the uterus as well as stimulation of milk ejection (6). The responses to VT are triggered in mammals by three distinct receptor subtypes (7) and are more complex [see (8), for review]. Two of these, the V1A and V1B subtypes, are clearly more like OTR and induce similar signal transduction responses primarily resulting in the release of Ca^2+^ as a second messenger. V1A is expressed in the brain where it regulates behavior and in vasculature where it induces vasoconstriction. The V1B receptor also regulates behavior and is the receptor that triggers the release of the hormone ACTH from the anterior pituitary and thereby the adrenal synthesis of cortisol and aldosterone. The V2 receptor, on the other hand, primarily stimulates adenylyl cyclase to synthesize cAMP and in kidney tubules this results in the reabsorption of water that reduces the volume of urine. V2 also stimulates the synthesis of coagulation factors.

In the light of the two ancestral vertebrate whole genome duplication (WGD) events (called 1R and 2R) before the radiation of jawed vertebrates (9, 10), it seemed likely that the four receptor genes had arisen from a common ancestral gene *via* two rounds of WGD, especially as they are located on four separate chromosomes in the human genome. However, when we analyzed the OT/VT receptor family in various jawed vertebrates, representing the major evolutionary lineages, we found firstly that jawed vertebrates have at least five OTR/VTR genes (11). Subsequently we concluded that that there are no less than six distinct receptor genes, with clear evidence of individual gene losses in some lineages (12).

The most parsimonious explanation for the extant repertoire of six OTR/VTR genes in jawed vertebrates that is compatible with sequence-based phylogeny, species distribution and chromosomal locations, is that an ancestral syntenic receptor pair was quadrupled in 1R/2R, whereupon two genes were lost before the jawed vertebrate radiation (12). This scenario was strongly supported by the four-fold symmetry of the chromosomal regions shown by a large number of adjacent gene families, demonstrating that not only the ancestral receptor gene pair had been quadrupled, but in fact also a large chromosomal region harboring a large number of other gene families. The species distribution and sequence phylogenies of these neighboring gene families were consistent with this chromosome quadruplication arising concomitantly with many other chromosome quartets, thus the two WGD events before the jawed vertebrate radiation.

Nevertheless, two more recent reports have questioned this parsimonious double WGD scheme and have proposed other scenarios. One study that also included lamprey OTR/VTR sequences concluded that the analyses supported one WGD event that was followed by “chromosomal or segmental duplications and translocations” in the jawed vertebrate lineage (13). This conclusion was repeated more recently in a book chapter from the same group (14). The most recent report on the evolution of OTR/VTR genes favored one WGD event followed by large segmental duplications (4).

Here, we have extended our previous report (12) including both OTR/VTR sequence data as well as chromosomal synteny analyses from newer high-coverage genome assemblies representing a wider selection of vertebrate lineages. Our analyses add strong support for four-fold symmetry, that is chromosome quadruplication, in the same time window as the two WGD events early in vertebrate evolution. Thus, the evolution of the OTR/VTR gene family can be most parsimoniously explained by two WGD events before the radiation of jawed vertebrates.

## 2 Results

### 2.1 Updated Phylogeny of Jawed Vertebrate OTR/VTR Genes

**Figure 1:**
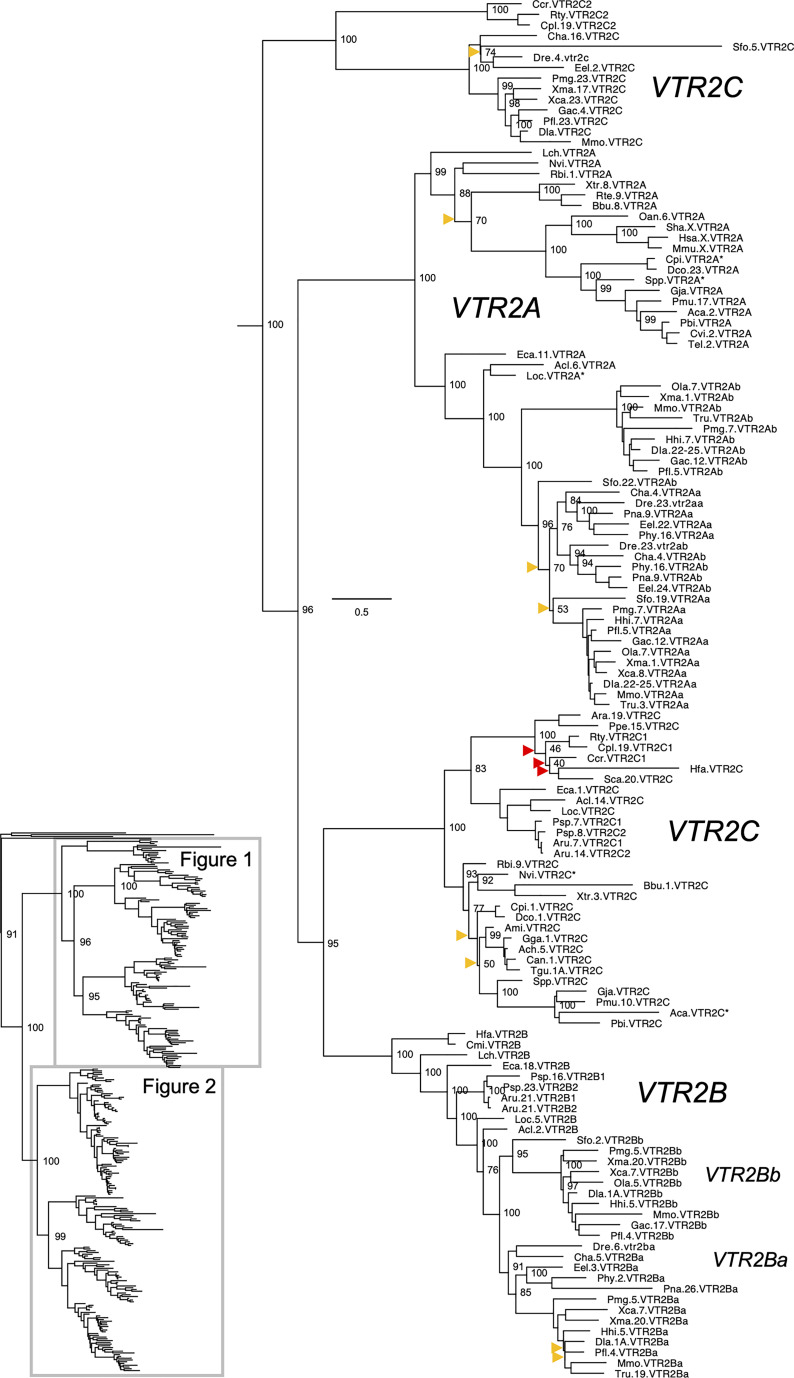
Maximum likelihood phylogeny of VTR2A, VTR2B and VTR2C genes. Inset: Placement of data shown in and within the OTR/VTR phylogeny. Phylogeny constructed with IQ-TREE supported by 100 iterations of ultrafast bootstrapping (UFBoot). Weakly supported nodes are indicated by yellow (<75 %) and red (<50 %) arrowheads. Some node support values for shallow nodes have been omitted for visual clarity. Sequence names include species abbreviations followed by chromosome/linkage group designations (where available) and gene symbols. Species abbreviations are listed in **Table 1**. Asterisks indicate partial sequences. The phylogeny was rooted with the common octopus (Octopus vulgaris) cephalotocin (*CTR1*, *CTR2*) and octopressin (*OPR*) receptor sequences.

**Figure 2:**
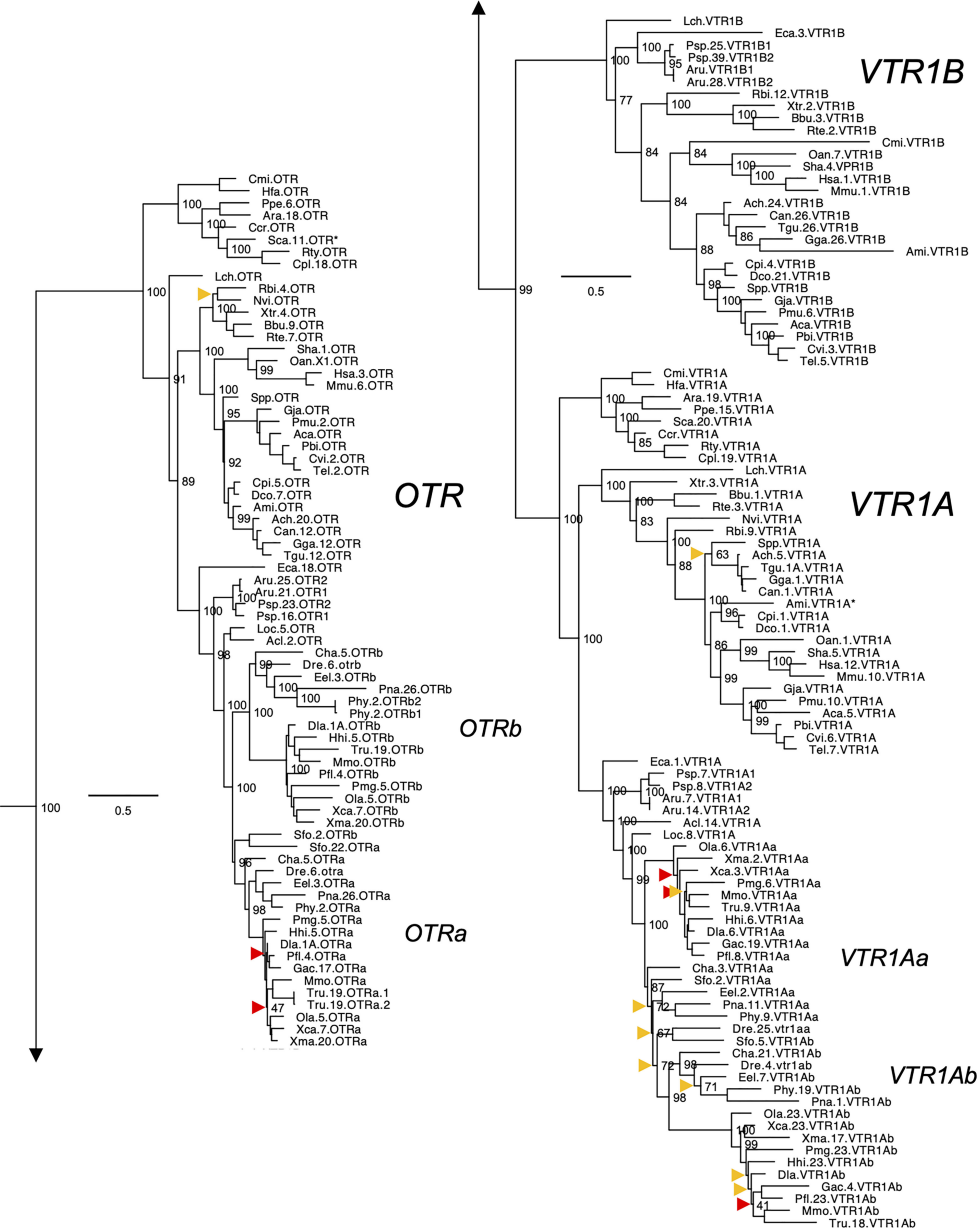
Maximum likelihood phylogeny of VTR1A, VTR1B and OTR genes. Inset: Placements of the *VTR2A*, *VTR2B* and *VTR2C* branches within the full OTR/VTR phylogeny. See caption for more details.

Our full updated maximum likelihood phylogeny of the OTR/VTR gene family, including all 88 analyzed species and 519 sequences, has been deposited in Figshare (see **Supplementary Material section**). A smaller phylogeny including 55 species (**Table 1**) is shown in **Figures 1** and **2**. The smaller phylogeny, as well as the alignments underlying both phylogenies, have also been deposited in Figshare. Our conclusions are supported by both phylogenies. We have used the gene nomenclature *VTR1A* (*AVPR1A*), *VTR1B* (*AVPR1B*), *OTR* (*OXTR*), *VTR2A* (*AVPR2*), *VTR2B* and *VTR2C*.

**Table 1:** Species and genome assembly information for species shown in **Figures 1** and **2**.

Abbreviation	Genus and species	Common name	Genome assembly
Hsa	*Homo sapiens*	Human	GRCh38.p13
Mmu	*Mus musculus*	Mouse	GRCm39
Sha	*Sarcophilus harrisii*	Tasmanian devil	mSarHar1.11
Oan	*Ornithorhynchus anatinus*	Platypus	mOrnAna1.pri.v4
Gga	*Gallus gallus*	Chicken	bGalGal1
Can	*Calypte anna*	Anna’s hummingbird	bCalAnn1_v1.p
Ach	*Aquila chrysaetos chrysaetos*	European golden eagle	bAquChr1.2
Tgu	*Taeniopygia guttata*	Zebra finch	bTaeGut2.pat.W.v2
Ami	*Alligator mississippiensis*	American alligator	ASM28112v4
Cpi	*Chrysemys picta bellii*	Western painted turtle	Chrysemys_picta_BioNano-3.0.4
Dco	*Dermochelys coriacea*	Leatherback sea turtle	rDerCor1.pri.v3
Spp	*Sphenodon punctatus*	Tuatara	ASM311381v1
Gja	*Gekko japonicus*	Schlegel’s Japanese gecko	Gekko_japonicus_V1.1
Pmu	*Podarcis muralis*	Common wall lizard	PodMur_1.0
Aca	*Anolis carolinensis*	Carolina anole lizard	AnoCar2.0
Pbi	*Python bivittatus*	Burmese python	Python_molurus_bivittatus-5.0.2
Cvi	*Crotalus viridis*	Western rattlesnake	UTA_CroVir_3.0
Tel	*Thamnophis elegans*	Western terrestrial garter snake	rThaEle1.pri
Rbi	*Rhinatrema bivittatum*	Two-lined caecilian	aRhiBiv1.1
Nvi	*Notophthalmus viridescens*	Eastern newt	reference_transcripts_v2
Xtr	*Xenopus tropicalis*	Western clawed frog	UCB_Xtro_10.0
Bbu	*Bufo bufo*	Common toad	aBufBuf1.1
Rte	*Rana temporaria*	European common frog	aRanTem1
Lch	*Latimeria chalumnae*	Coelacanth	LatCha1
Eca	*Erpetoichthys calabaricus*	Reedfish	fErpCal1.1
Psp	*Polyodon spathula*	American paddlefish	ASM1765450v1
Aru	*Acipenser ruthenus*	Sterlet	ASM1064508v1
Loc	*Lepisosteus oculatus*	Spotted gar	LepOcu1
Acl	*Amia calva*	Bowfin	AmiCal1
Sfo	*Scleropages formosus*	Asian arowana	fSclFor1.1
Cha	*Clupea harengus*	Atlantic herring	Ch_v2.0.2
Dre	*Danio rerio*	Zebrafish	GRCz11
Pna	*Pygocentrus nattereri*	Red-bellied piranha	fPygNat1.pri
Eel	*Electrophorus electricus*	Electric eel	fEleEle1.pri
Phy	*Pangasiodon hypophthalmus*	Striped catfish	GENO_Phyp_1.0
Pmg	*Periophthalmus magnuspinnatus*	Big-finned mudskipper	fPerMag1.pri
Hhi	*Hippoglossus hippoglossus*	Atlantic halibut	fHipHip1.pri
Ola	*Oryzias latipes*	Japanese medaka	ASM223467v1
Xca	*Xenentodon cancila*	Freshwater garfish	fXenCan1.pri
Xma	*Xiphophorus maculatus*	Southern platyfish	X_maculatus-5.0-male
Gac	*Gasterosteus aculeatus*	Three-spined stickleback	BROAD S1
Pfl	*Perca fluviatilis*	European perch	GENO_Pfluv_1.0
Dla	*Dicentrarchus labrax*	European sea bass	seabass_V1.0
Mmo	*Mola mola*	Ocean sunfish	ASM169857v1
Tru	*Takifugu rubripes*	Japanese pufferfish	fTakRub1.2
Cmi	*Callorhinchus milii*	Elephant shark	Callorhinchus_milii-6.1.3
Haf	*Hydrolagus affinis*	Small-eyed rabbitfish	UP_Haf
Ara	*Amblyraja radiata*	Thorny skate	sAmbRad1.1.pri
Ppe	*Pristis pectinata*	Smalltooth sawfish	sPriPec2.pri
Rty	*Rhincodon typus*	Whale shark	ASM164234v2
Cpl	*Chiloscyllium plagiosum*	Whitespotted bambooshark	ASM401019v1
Ccr	*Carcharodon carcharias*	White shark	ASM360424v1
Sca	*Scyliorhinus canicula*	Small-spotted catshark	sScyCan1.2
Cin	*Ciona intestinalis*	Vase tunicate	KH
Ovu	*Octopus vulgaris*	Common octopus	

The updated phylogenies are consistent with our previously published analyses (12, 15), showing 6 ancestral subtypes *VTR2A*, *VTR2B* and *VTR2C* (**Figure 1**) as well as *VTR1A*, *VTR1B* and *OTR* (**Figure 2**) diverging early in jawed vertebrate evolution. As in previous analyses, the *VTR2C* genes form a paraphyletic group, with the *VTR2C* sequences from teleost fishes (Teleostei) as well as some shark (Selachimorpha) *VTR2C* sequences clustering at the base of the *VTR2A*, *VTR2B* and the main *VTR2C* branches (**Figure 1**). Nonetheless, conserved synteny as well as exon/intron structures and amino acid sequence features support the identity of this seemingly basal branch as *VTR2C*. All other OTR/VTR subtypes are well-supported and, in general, follow the known and accepted phylogeny of jawed vertebrate groups. As in previous analyses, the coelacanth (*Latimeria chalumnae*) OTR/VTR sequences in some cases diverge from the expected phylogenetic position, see the *VTR1B* and *OTR* branches in **Figure 2**. Other discrepancies in our current phylogenies include the following:

- The two-lined caecilian (*Rhinatrema bivittatum*) sequences often cluster at the base of the full tetrapod branches rather than at the base of the amphibian branches. Additionally, the amphibian *VTR1A* branch could not be reconstructed (**Figure 2**).
- Our phylogenies often fail to reconstruct Archosauria (including turtles (16), crocodilians and birds) on one side and Lepidosauria (tuatara, lizards and snakes) on the other, with the tuatara (*Sphenodon punctatus*) and turtle (Testudines) sequences often occupying ambiguous positions.
- The spotted gar (*Lepiososteus oculatus*) and bowfin (*Amia calva*) (both Holostei) *VTR1A* and *VTR2B* sequences do not cluster together, however they occupy the expected positions as immediate outgroups to the corresponding teleost branches.
- The Asian arowana (*Scleropages formosus*) sequences often occupy ambiguous positions within teleosts, rather than diverging at the base of teleosts. For example, the Asian arowana *VTR2C* sequence appears within the otocephalan clade rather than at the base of the teleost branch, likely due to a long-branch artefact (**Figure 1**). In general, sequences from basally radiating teleosts occupy ambiguous positions in the phylogenies with respect to 3R-generated -*a* and *-b* branches. Likely due to the short time window between 3R and their radiations and uneven divergence rates for the resulting co-orthologs. For these reasons we carried out an additional conserved synteny analysis in the Asian arowana (described in section 4.3 below), which supports the assigned gene names shown in **Figures 1** and **2**.

Aside from these minor discrepancies, both phylogenies are generally well-supported and well-resolved, and allowed us to draw more detailed conclusions about the repertoires of OTR/VTR subtype genes in different vertebrate lineages than before.

### 2.2 Key Lineages in the Updated OTR/VTR Phylogeny

#### 2.2.1 Basal Ray-Finned Fishes

Key species added to our updated phylogenies include the basal ray-finned fishes (Actinopterygii) reedfish (*Erpetoichthys calabaricus*) and gray bichir (*Polypterus senegalus*) (both Polypteriformes), as well as American paddlefish (*Polyodon spathula*) and sterlet (*Acipenser ruthenus*) (both Acipenseriformes). In addition to including these species in our updated phylogenies, we included the reedfish genome in our updated conserved synteny analyses, as described below. We found that the reedfish genome contains the full ancestral jawed vertebrate complement of OTR/VTR genes, and that these reedfish genes, in all cases but one, cluster in the expected positions in our phylogenies (**Figures 1** and **2**). The only exception is the *VTR1B* sequence, which clusters together with the American paddlefish and sterlet *VTR1B* sequences rather than at the base of the ray-finned fish branch (**Figure 2**). We could also find the full complement of genes in the gray bichir genome (see *Data Availability Statement* below for access to the full phylogeny). We could identify duplicates of the *VTR1A*, *VTR1B*, *OTR*, *VTR2B* and *VTR2C* genes in both the American paddlefish and sterlet (**Figures 1** and **2**), with each pair located on chromosome pairs that are consistent with the respective independent WGDs in each of the two lineages (17, 18). The only exceptions are the sterlet *VTR1B1* gene, which is located on an unplaced scaffold, and the sterlet *VTR2B1* and *VTR2B2* duplicates, which are both located on chromosome 21. Furthermore, our phylogenies are also consistent with independent WGDs in the American paddlefish and the sterlet. The age of the American paddlefish WGD has been estimated at 50 million years ago (MYA) (18) and the sterlet WGD has been estimated at both 180 MYA (17) and 21.3 MYA (19). We found that both the American paddlefish and sterlet genomes lacked a *VTR2A* gene, suggesting an ancestral gene loss in sturgeons (Acipenseriformes).

#### 2.2.2 Cartilaginous Fishes

A key addition for our analyses is the inclusion of seven cartilaginous fish (Chondrichthyes) species in addition to the elephant shark (*Callorhinchus milii*) (**Table 1**), thus representing all three major cartilaginous fish lineages in our phylogenies. We also added the thorny skate (*Amblyraja radiata*) genome to our updated conserved synteny analyses, as described below. We could only identify *VTR1A*, *OTR* and *VTR2C* in the thorny skate, smalltooth sawfish (*Pristis pectinata*) (also Batoidea) and shark genomes. The *VTR1A* and *OTR* sequences cluster in the expected phylogenetic positions, while the cartilaginous fish *VTR2C* sequences cluster with the basal ray-finned fish *VTR2C* sequences rather than at the base of the jawed vertebrate *VTR2C* branch. In addition, some of the shark genomes had an additional local duplicate of *VTR2C*, which we have called *VTR2C2*. Notably, the shark *VTR2C2* sequences cluster with the teleost *VTR2C* sequences at the base of the V2R branch. This is described further in section 2.4.3 below. We have previously shown that the elephant shark genome contains 5 of the ancestral jawed vertebrate genes, lacking only *VTR2A* (12). Our current phylogenetic analyses support the loss of *VTR2A* at the base of extant cartilaginous fishes, with further losses of *VTR1B* and *VTR2B* at the base of Elasmobranchii.

We searched for the missing genes *VTR1B*, *VTR2A* and *VTR2B* in several available transcriptome assemblies from cartilaginous fish species: the zebra bullhead shark (*Heterodontus zebra*) (20), ocellate spot skate (*Okamejei kenojei*) (21), blue shark (*Prionace glauca*) (22), elephant shark (23), winter skate (*Leucoraja ocellata*) (24), and small-spotted catshark (*Scyliorhinus canicula*) (25). However, we could not identify putative *VTR1B*, *VTR2A* or *VTR2B* transcripts in these datasets. We could only identify partial *VTR1A* and *OTR* transcripts in the zebra bullhead shark and ocellate spot skate transcriptomes, and a partial *VTR1A* transcript in the winter skate transcriptome.

#### 2.2.3 Other Notable Gene Losses

We found that *VTR2A* was missing from the American alligator (*Alligator mississippiensis*) genome as well as from all analyzed avian genomes. However, it is present in both analyzed turtle species: the Western painted turtle (*Chrysemys picta bellii*) and the leatherback sea turtle (*Dermochelys coriacea*) (**Figure 1**). This suggests a loss of *VTR2A* in an archosaurian ancestor of birds and crocodilians.

We could not identify *VTR2C* in the Western rattlesnake (*Crotalus viridis*), Indian cobra (*Naja naja*) and Western terrestrial garter snake (*Thamnophis elegans*) genomes. However, it is found in the genome of the Burmese python (*Python bivittatus*), which represents a more basal snake lineage. This suggests a loss of *VTR2C* from the lineage leading to caenophidian snakes, which represent more than 80 % of extant snake species (26).

Some teleosts lack duplicate *VTR2B* genes. Otocephalan teleosts seem to have lost the *VTR2Bb* duplicate – It could not be identified in the Atlantic herring (*Clupea harengus*), zebrafish (*Danio rerio*), red-bellied piranha (*Pygocentrus nattereri*), electric eel (*Electrophorus electricus*) or striped catfish (*Pangasiodon hypophthalmus*) genomes. The *VTR2Bb* duplicate is also missing from the green spotted pufferfish (*Tetraodon nigroviridis*) and Japanese pufferfish (*Takifugu rubripes*), however both duplicates are present in the ocean sunfish (*Mola mola*) (all Tetraodontiformes). It is also missing from the Atlantic cod (*Gadus morhua*) and the closely related walleye pollock (*Gadus chalcogrammus*). Conversely, the *VTR2Ba* duplicate could not be identified in the Asian or African arowana genomes, the Japanese medaka (*Oryzias latipes*) genome (although both *VTR2B* duplicates are present in the freshwater garfish (*Xenentodon cancila*), also Beloniformes), nor in the three-spined stickleback (*Gasterosteus aculeatus*) or the closely related *Gasterosteus nipponicus* and nine-spined stickleback (*Pungitius pungitius*) genomes. Both *VTR2B* duplicates are absent from the Northern pike (*Esox lucius*) genome, as well as from two other species in the genus *Esox* whose genomes are available (not shown herein).

We could not identify *VTR2C* genes in the red-bellied piranha or striped catfish within Otocephala. It is also missing from the Japanese medaka as well as from the five other available genomes from the genus *Oryzias* (not shown herein), however, we could identify it in the freshwater garfish (also in the order Beloniformes). *VTR2C* is also seemingly missing from the spotted green pufferfish and Japanese pufferfish genomes. Searches for *VTR2C* in five other available genomes from Tetraodontiformes (not shown herein) also returned negative results, however, it is present in the ocean sunfish (also Tetraodontiformes) on the same genomic scaffold as the *VTR1Ab* gene. Additionally, *VTR2C* could not be identified in the Atlantic cod (*Gadus morhua*), nor the closely related walleye pollock (*Gadus chalcogrammus*), the Atlantic halibut (*Hippoglossus hippoglossus*), or the lumpfish (*Cyclopterus lumpus*).

*VTR1Ab* and *VTR2C*, which are located on the same chromosome in other teleost species, could not be identified in the greater pipefish (*Syngnathus acus*) genome, nor in the big-belly seahorse (*Hippocampus abdominalis*) (both Syngnathiformes).

#### 2.2.4 Cyclostome OTR/VTR Family Members

Like in the recent study by Theofanopoulou et al. (4), we included sea lamprey (*Petromyzon marinus*) and inshore hagfish (*Eptatretus burgeri*) OTR/VTR sequences in our full phylogeny (see *Data Availability Statement* below for access to the full phylogeny). We could replicate the findings in Theofanopoulou et al. (4), regarding the phylogenetic positions of these sequences within the vertebrate OTR/VTR phylogeny. The phylogeny suggests that cyclostomes have orthologs of *OTR* and *VTR2C*, however there is an additional VTR subtype of unclear relationship that Theofanopoulou et al. (4) call *VTR1A* but that in fact clusters at the base of *VTR1A*, *VTR1B* and *OTR* in both their phylogeny and ours. Additionally Theofanopoulou et al. (4) identify one of the inshore hagfish sequences as *VTR1A* whereas our phylogeny places this sequences at the base of the *OTR* branch. However, we consider these results as interim in wait for more careful conserved synteny analyses which are underway in the latest sea lamprey genome assembly. The recent report that cyclostomes have undergone an independent hexaploidization (27) means that gene relationships have to be carefully analyzed, considering synteny as well as phylogeny.

### 2.3 Vertebrate Oxytocin and Vasotocin Receptor Gene Features

#### 2.3.1 Exon/intron Organization

**Figure 3:**
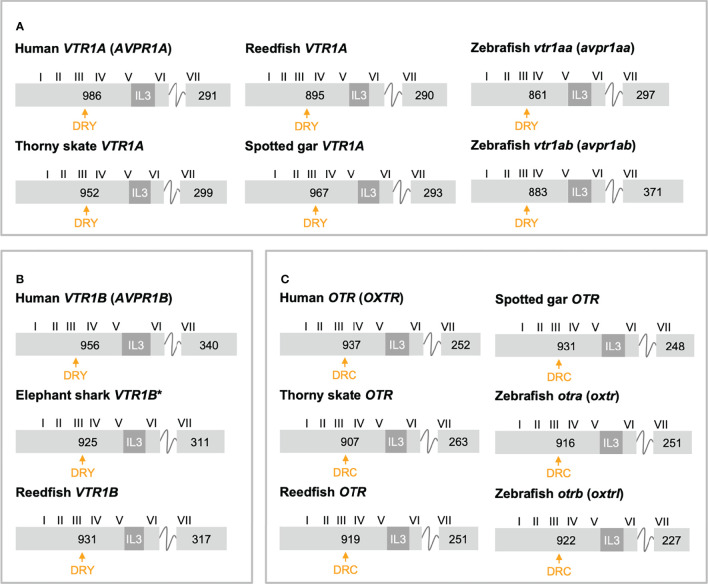
Exon/intron structures of human, thorny skate, reedfish, spotted gar and zebrafish VTR1A **(A)**, VTR1B **(B)** and OTR **(C)** genes. Additional representative genes are marked with an asterisk. Only coding exons are shown. Exons are drawn to scale and exon lengths are given in base-pairs. Roman numerals designate the approximate locations of the transmembrane (TM) region-encoding sequences. The third intracellular loop (IL3)-encoding sequences are indicated in dark gray. The conserved DRY/DRC motifs are indicated by yellow arrows.

**Figure 4:**
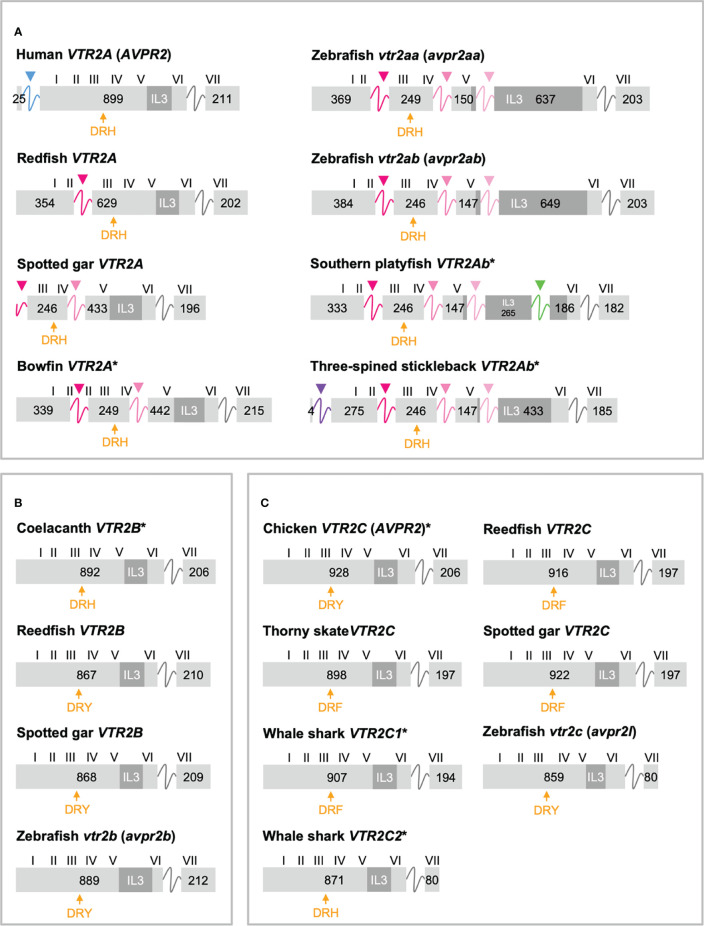
Exon/intron structures of human, thorny skate, reedfish, spotted gar and zebrafish VTR2A **(A)**, VTR2B **(B)** and VTR2C **(C)** genes. Additional representative genes are marked with an asterisk. See caption for details. Within the OTR/VTR gene family, only *VTR2A* genes (A) diverge from the conserved exon/intron structure due to intron insertions in several lineages. The blue arrowhead marks the first intron of the human *VTR2A* (*AVPR2*) gene (in blue), which is shared with all mammalian *VTR2A* genes. Pink arrowheads indicate introns inserted in ray-finned fish evolution between conserved Gln-Val (Q-V)-encoding codons. The green arrowhead indicates a predicted intron inserted into the IL3-encoding region in *VTR2Ab* genes from spiny-rayed fishes (Acanthomorpha). The purple arrowhead indicates a predicted N-terminal intron seen only in the three-spined and nine-spined stickleback *VTR2Ab* genes.

The exon/intron organizations of representative OTR/VTR genes analyzed in this study are shown in **Figure 3** (*VTR1A*, *VTR1B* and *OTR*) and **Figure 4** (*VTR2A*, *VTR2B* and *VTR2C*). An annotated rich text file document which shows the exon/intron boundaries overlaid on all analyzed sequences has been deposited in Figshare (see **Supplementary Material**). The human *VTR1A* (*AVPR1A*), *VTR1B* (*AVPR1B*) and *OTR* (*OXTR*) genes each comprises two coding exons, while the *VTR2A* (*AVPR2*) comprises three coding exons. The additional intron in the beginning of the human *VTR2A* gene coding region (28) (blue arrow in **Figure 4A**), arose in a mammalian ancestor, while the second intron is an ancestral intron conserved in vertebrate OTR/VTR genes. This basic exon/intron organization is present in all vertebrate OTR/VTR genes analyzed in this study, including those from basal ray-finned fishes, and cartilaginous fishes. However, there have been several waves of additional intron insertion in the *VTR2A* genes.

In a previous study (11) we reported that teleost *VTR2A* genes had 3 additional introns upstream of the conserved intron, all inserted between CAG (Gln, Q) and GTG/GTT (Val, V) codons. These introns are indicated with magenta/pink arrows in **Figure 4A**. With the additional basal ray-finned fish species analyzed in this study, we can present a more detailed timeline for these intron insertions. The first Q-V intron was likely inserted already in a ray-finned fish ancestor, as this intron is preserved in both the reedfish (**Figure 4A**) and gray bichir *VTR2A* genes. The second Q-V intron was inserted in a common ancestor of holosteans (spotted gar and bowfin in **Figure 4A**) and teleosts, at the latest. We could not identify *VTR2A* in the American paddlefish or sterlet, thus it is possible that this intron was inserted earlier, in a common ancestor of chondrostean and neopterygian fishes (Holostei and Teleostei). The last of the three Q-V introns was inserted early in teleost evolution, before the 3R WGD event, as both *VTR2Aa* and *VTR2Ab* duplicates have it.

A putative fourth intron interrupting the main intracellular loop 3 (IL3)-encoding exon could be identified at a conserved location in most spiny-rayed fish (Acanthomorpha) *VTR2Ab* genes (green arrowhead in **Figure 4A**). This “IL-3” intron could not be identified in the three-spined stickleback *VTR2Ab* gene, nor in that of the closely related nine-spined stickleback (*Pungitius pungitius*), suggesting an intron deletion in this lineage. Conversely, the three-spined and nine-spined stickleback *VTR2Ab* genes have a putative intron insertion in the N-terminal encoding exon (purple arrowhead in in **Figure 4A**). This putative intron is a prerequisite to predict a correct N-terminal signal peptide sequence for these genes.

#### 2.3.2 Elongated Intracellular Loop 3 in Teleost VTR2A

We also reported in a previous study (11) that teleost *VTR2A* genes seemed to have an elongated IL3-encoding exon, producing a notably elongated middle portion of this intracellular loop. However, at the time we could not identify conserved motifs within this elongated region due to the scarcity of sequences. In the present study, we have analyzed 68 teleost *VTR2Aa* and *VTR2Ab* sequences with elongated IL3 sequences and were able to identify several conserved, albeit short, sequence motifs, primarily based around conserved Pro (P) residues, and to some extent several conserved Tyr (Y) as well as Cys (C) residues. The elongated IL3 sequences are also rich in relatively well-conserved Ser (S) and Thr (T) residues. These features can be seen in the full sequence alignment that has been deposited in Figshare (see **Supplementary Material section**). We also performed predictions of intrinsically disordered regions for all teleost *VTR2A* amino acid sequences using IUPred2A (https://iupred2a.elte.hu/) (29, 30) and could detect a high degree of disorder spanning the length of the elongated IL3 sequences for all teleost *VTR2Aa* and *VTR2Ab* sequences (not shown herein).

#### 2.3.3 Shortened C-Terminal in Cartilaginous Fish and Teleost *VTR2C*

Another striking feature among the vertebrate OTR/VTR genes is the shortened C-terminal-encoding exon seen in teleost *VTR2C* genes as well as the shark *VTR2C2* genes (**Figure 4C**). Additionally, the teleost *VTR2C* and shark *VTR2C2* cluster together at the base of the V2R branch of our phylogenies (**Figure 1**). This phylogenetic position, as well as the similarly shortened C-terminal between the genes across a large evolutionary distance, seem to indicate that they are orthologous. This would mean that a seventh *VTR2C*-like gene arose before the jawed vertebrate radiation and subsequently was lost independently from all jawed vertebrate lineages except sharks and teleosts. A more parsimonious interpretation is that a shark-specific local duplicate of the *VTR2C* gene and the teleost *VTR2C* gene independently acquired premature stop codons at roughly the same positions, shortening their C-terminal tails. This, along with the relative scarcity of shark *VTR2C2* and teleost *VTR2C* genes, likely creates the phylogenetic artefact seen in our phylogenies. However, the possibility that these genes represent a seventh ancestral OTR/VTR receptor subtype gene that arose through a local duplication of the *VTR2C* gene (as indicated by the chromosomal locations in cartilaginous fishes), cannot be completely discounted.

### 2.4 Conserved Synteny Analyses

#### 2.4.1 Involvement of Early Vertebrate WGDs (1R/2R)

**Figure 5:**
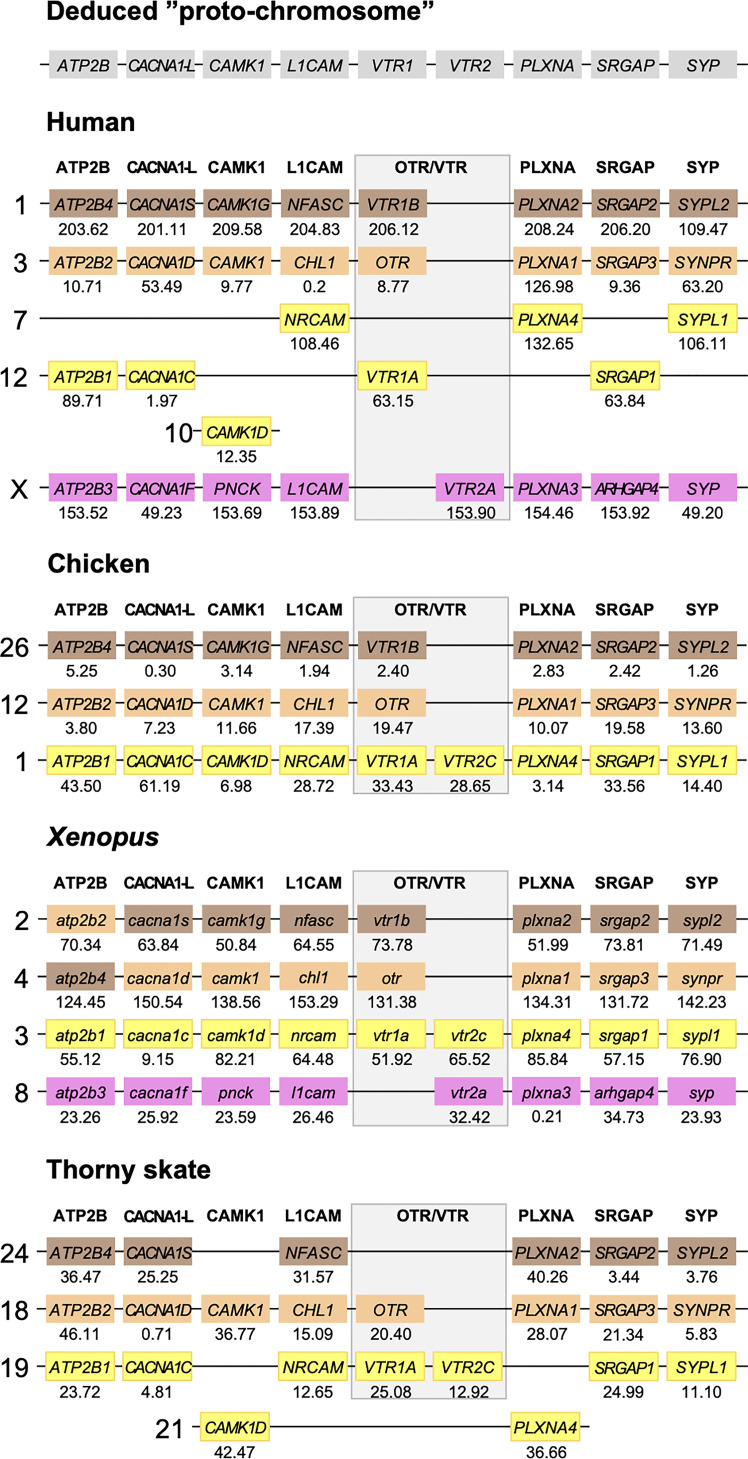
Conserved synteny in OTR/VTR gene-bearing chromosome regions in the human, chicken, Xenopus and thorny skate genomes. *Xenopus tropicalis* gene symbols are in lower case, following the gene nomenclature convention for this species.

**Figure 6:**
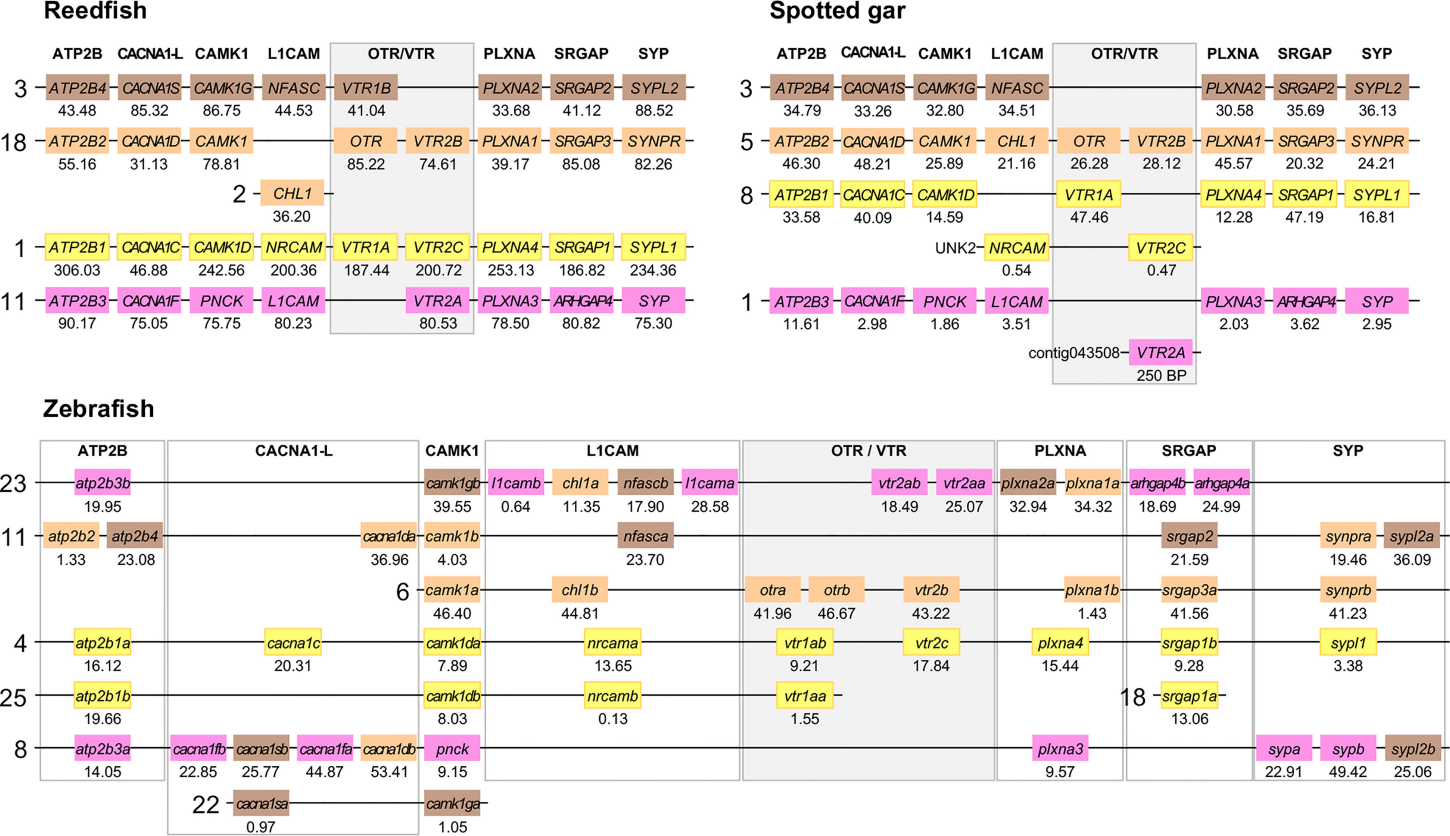
Conserved synteny in OTR/VTR gene-bearing chromosome regions in the reedfish, spotted gar and zebrafish genomes. We suggest the nomenclature *OTRa* and *OTRb* for the teleost isotocin receptor gene duplicates, and VTR2a and VTR2b for the vasotocin receptor VTR2B gene duplicates. Zebrafish gene symbols are in lower case, following the gene nomenclature convention for this species.

Herein, we have re-analyzed the chromosomal locations of seven representative neighboring gene families (**Table 2**) from a previous analysis in the human, chicken (*Gallus gallus*), spotted gar and zebrafish genomes (12), and added chromosomal information from newer high-coverage genome assemblies from species that occupy key phylogenetic positions: the polypteriform fish reedfish, the cartilaginous fish thorny skate and a more recent high-quality assembly of the Western clawed frog (*Xenopus tropicalis*) genome. ﻿To investigate the involvement of WGDs, the chromosomal locations of all neighboring gene family members were recorded and compared across species. The identified chromosome locations in the human, chicken, Western clawed frog and thorny skate are shown in **Figure 5** while those in the reedfish, spotted gar and zebrafish genomes are shown in **Figure 6**. All chromosome locations and database identifiers for the neighboring genes are available in Figshare (see **Supplementary Material**).

**Table 2:** Neighboring gene families.

Abbreviation	Description	Genes
ATP2B	ATPase plasma membrane Ca^2+^ transporting protein B	*ATP2B1*, *ATP2B2*, *ATP2B3*, *ATP2B4*
CACNA1-L	Voltage-gated Ca^2+^ channel alpha-1 subunit, L-type	*CACNA1C*, *CACNA1D*, *CACNA1F*, *CACNA1S*
CAMK1	Calcium/calmodulin dependent protein kinase 1	*CAMK1*, *CAMK1D*, *CAMK1G*, *PNCK*
L1CAM	L1-related cell-adhesion molecule	*CHL1*, *L1CAM*, *NFASC*, *NRCAM*
PLXNA	Plexin A	*PLXNA1*, *PLXNA2*, *PLXNA3*, *PLXNA4*
SRGAP	SLIT-ROBO Rho GTPase activating protein	*SRGAP1*, *SRGAP2*, *SRGAP3*, *ARHGAP4*
SYP	Synaptophysin/synaptoporin family	*SYP*, *SYPL1*, *SYPL2*, *SYNPR*

The results in the human, chicken, spotted gar and zebrafish genomes essentially replicate our previous results from (12). There are small changes in location for each individual gene, however this is expected considering the updates in genome assembly version between 2013 and 2021, and in all relevant aspects the identified chromosome regions are the same. For the spotted gar, we could identify several neighboring genes that we could not identify previously, namely *ATP2B4*, *CACNA1D*, *CACNA1F*, *PNCK*, *CHL1*, *L1CAM*, *PLXNA3*, *ARHGAP4*, *SRGAP2*, *SYPL1* and *SYPL2* (**Figure 6**). Most of these are from the *VTR2A*-bearing chromosome region. The identified chromosomal regions in the Western clawed frog genome support our previous findings and all neighboring family genes could be identified (**Figure 5**), albeit with a small translocation of the *atp2b2* and *atp2b4* genes between the paralogous chromosome regions.

We could identify three of the four paralogous chromosome regions in the thorny skate genome. Notably, we could not identify any of the neighboring genes in the *VTR2A*-bearing region, nor *CAMK1G* in the region that used to have *VTR1B* (**Figure 5**). In addition, there seems to have been a linkage break in the *VTR1A-VTR2C*-bearing region between chromosome 19 and 21. In any case, the conserved synteny analysis in the thorny skate provides us with the earliest relative time point for the *VTR1B*, *OTR-VTR2B* and *VTR1A-VTR2C*-bearing chromosome regions, before the divergence of cartilaginous and bony fishes (Osteichthyes).

We could identify all four paralogous chromosome regions in the reedfish genome, including all neighboring family genes (**Figure 6**). There seems to have been a linkage break in the *OTR-VTR2B*-bearing chromosome region, with the *CHL1* gene located on chromosome 2 rather than on chromosome 18. Nevertheless, the conservation of synteny in the reedfish genome provides us with a relative time point close to the divergence point between ray-finned fishes and lobe-finned fishes (Sarcopterygii) for all four paralogous OTR/VTR gene-bearing chromosome regions.

#### 2.4.2 Involvement of Early Teleost WGD (3R)

The conserved synteny pattern in the zebrafish genome reveals the extensive genome rearrangements that the teleost lineage underwent both prior to and following the 3R WGD event (31), which obscure the 1:2 correspondence between pre- and post-WGD chromosome regions (**Figure 6**). In most teleost genomes that have been analyzed so far, the duplicate *OTRa*/*OTRb* and *VTR2Aa*/*VTR2Ab* gene pairs are located on the same chromosomes, which prevents further analysis to elucidate the involvement of the 3R WGD. We found that the *OTRa*/*OTRb* and *VTR2Aa*/*VTR2Ab* gene pairs were located on separate chromosomes in the European eel (*Anguilla anguilla*) and Indo-Pacific tarpon (*Megalops cyprinoides*) (both Elopomorpha) as well as the Asian and African arowanas (*Scleropages formosus* and *Heterotis niloticus*, Osteoglossomorpha). These species represent the two basal-most lineages of teleosts, which likely diverged before genome rearrangements translocated the gene pairs to the same chromosomes.

**Figure 7:**
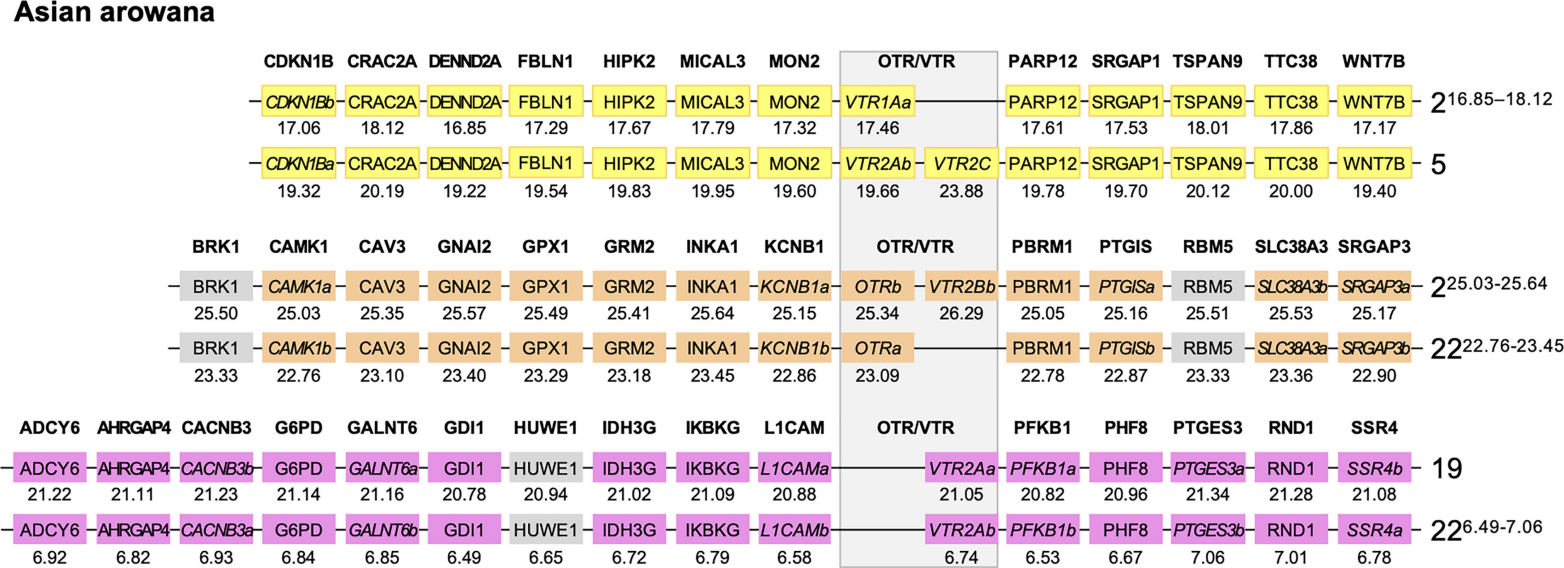
Conserved synteny in OTR/VTR gene-bearing chromosome regions in the Asian arowana genome shows evidence of duplications in the teleost whole genome duplication (3R). All identified gene pairs, apart from the *BRK1*, *RBM5* and *HUWE1* gene pairs (in gray), showed divergence early in teleost evolution, rooted by a single spotted gar and/or reedfish ortholog in the corresponding Ensembl gene trees. Where the orthology relationships to the zebrafish genes was clear in the Ensembl gene trees, gene names with “a” and “b”-designations have been suggested. Where there was only one zebrafish ortholog, this was designated the “a”-duplicate.

We used the Asian arowana *VTR1Aa*, *VTR1Ab*, *OTRa*, *OTRb*, *VTR2Aa*, *VTR2Ab* and *VTR2Bb* gene locations to identify patterns of conserved synteny in Genomicus (https://www.genomicus.bio.ens.psl.eu/genomicus-100.01). We identified 12 gene pairs neighboring the *VTR1Aa* and *VTR1Ab* genes on chromosomes 2 and 5; 13 gene pairs neighboring the *OTRa* and *OTRb* genes on chromosomes 2 and 22; and 15 gene pairs neighboring the *VTR2Aa* and *VTR2Ab* genes on chromosomes 19 and 22 (**Figure 7**). The identified conserved synteny blocks on chromosome 2 around *VTR1Aa* and *OTRb* do not overlap, nor do the conserved synteny blocks on chromosome 22 around *OTRa* and *VTR2Ab*. We analyzed the corresponding Ensembl gene trees for each gene pair and could determine that all identified gene pairs apart from the *BRK1*, *RBM5* and *HUWE1* gene pairs (in gray in **Figure 7**) showed divergence early in teleost evolution, rooted by a single spotted gar and/or reedfish ortholog, which indicates duplications through the 3R WGD.

## 3 Discussion

### 3.1 Update of Analyses

To extend our previous analyses of jawed vertebrate chromosomal regions for the OTR/VTR family, we have added information from newer high-coverage genome assemblies, particularly genomes that are distantly related to those that were available when we published our previous report describing the four-fold chromosomal similarity (12). At that time, we focused our analyses on chromosomal regions in the human, chicken, spotted gar and zebrafish genomes, and produced a phylogeny that also included OTR/VTR sequence information from other jawed vertebrates, namely mouse (*Mus musculus*), gray short-tailed opossum (*Monodelphis domestica*), Carolina anole lizard (*Anolis carolinensis*), Western clawed frog, coelacanth, several teleosts, and the holocephalan cartilaginous fish elephant shark.

Here, we have added OTR/VTR sequence information from a larger number of vertebrate species, as well as chromosomal regions from the polypteriform fish reedfish, representing an even earlier-diverging branch of the ray-finned fishes, the cartilaginous fish thorny skate as well as a more recent high-quality assembly of the Western clawed frog genome. We focused on species with key phylogenetic and evolutionary positions as well as high-coverage genome assemblies mapped to chromosomes or pseudochromosomes. In particular, we benefited from the recent advancements in the sequencing of cartilaginous fish genomes (32–35) as well as the advancement of the Vertebrate Genomes Project (36). This study includes two additional mammalian species, including platypus (*Ornithorhynchus anatinus*), nine additional avian species representing a wide segment of the extant avian diversity, nine additional reptilian species, four additional amphibian species, the gray bichir, reedfish, American paddlefish (18) and sterlet (17), which occupy key positions at the base of ray-finned fish evolution, ten additional teleost species, seven additional cartilaginous fish species representing all three major lineages, as well as the cyclostomes inshore hagfish (*Eptatretus burgeri*) (37), Pacific lamprey (*Entosphenus tridentatus*) (38) and sea lamprey (*Petromyzon marinus*). The full list of species, including genome assembly and database information has been deposited in Figshare (see **Supplementary Material section**), and a subset is shown in **Table 1**.

Updating the data with newly available assemblies also allowed us to assign chromosome locations to previously unassigned genes, as for the Western clawed frog genes and several of the three-spined stickleback (*Gasterosteus aculeatus*) genes, or to correct previously incomplete gene predictions. We also found that many of the NCBI gene models we identified for this study matched our manually curated predictions from the previous study (12), lending some validity to the manual curation process we had employed. All chromosome locations and gene/sequence prediction notes are available in Figshare (see **Supplementary Material**).

We constructed a full maximum likelihood phylogeny based on all 519 identified OTR/VTR sequences from 88 species (see **Supplementary Material**), as well as a smaller phylogeny shown in **Figures 1** and **2** based on the 55 representative species in **Table 1**. Both phylogenies support the emergence of the *VTR1A*, *VTR1B*, *OTR*, *VTR2A*, *VTR2B* and *VTR2C* genes early in vertebrate evolution, prior to the radiation of jawed vertebrates. We could identify all OTR/VTR subtype genes in cartilaginous fishes, except for *VTR2A*. We do not interpret this fact as support for the view that *VTR2A* arose in a bony fish (Osteichthyes) ancestor, millions of years after the emergence of *VTR2A* and *VTR2B*. Assuming an even rate of sequence evolution, our phylogenies suggest that the *VTR2A*, *VTR2B* and *VTR2C* genes arose concomitantly, and thus that the loss of *VTR2A* in cartilaginous fishes is a subsequent and independent event. This view is also supported by our chromosomal analyses of conserved synteny, discussed below.

### 3.2 Evolutionary Scenario for the Jawed Vertebrate OTR/VTR Gene Family

**Figure 8:**
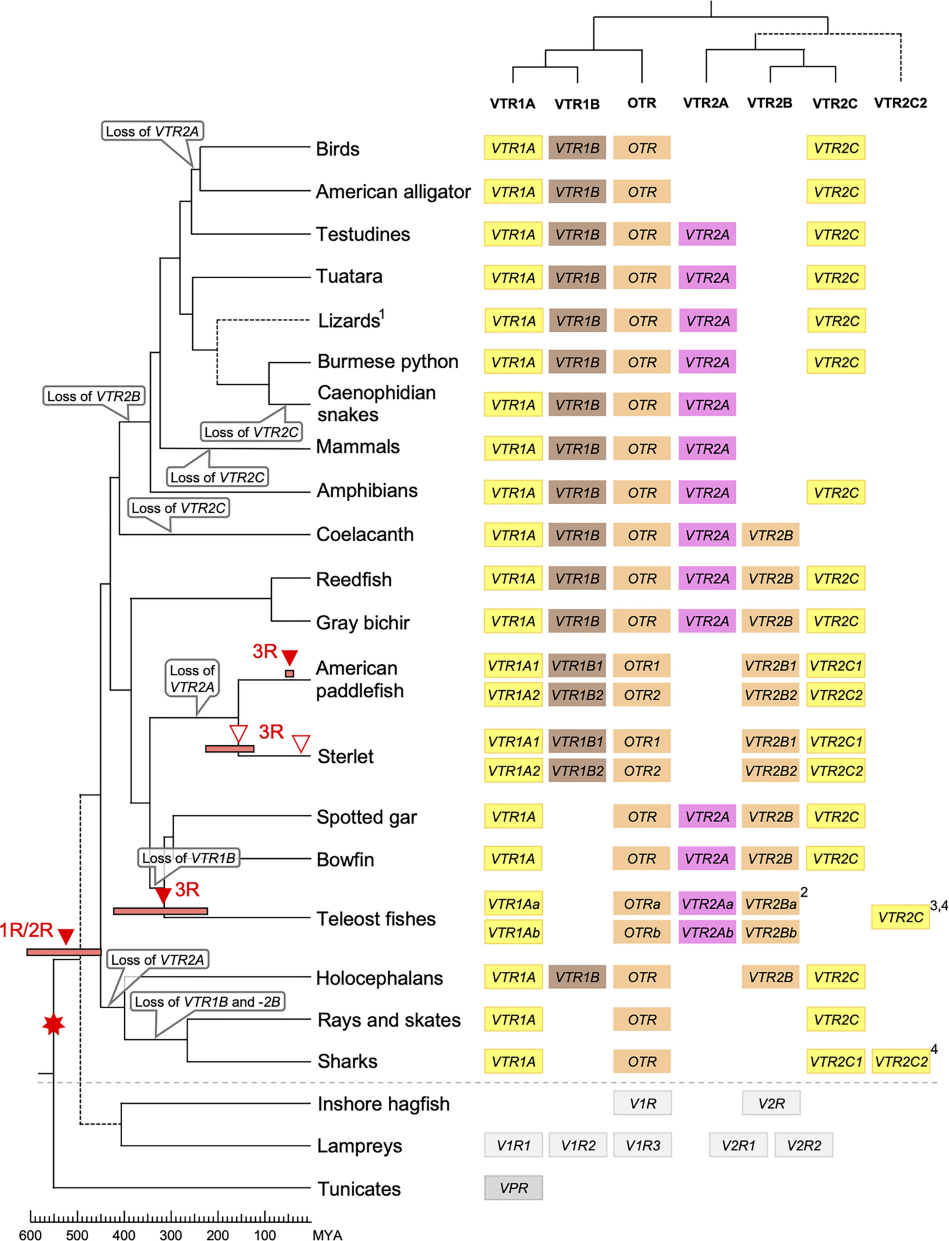
Proposed evolution of OTR/VTR genes and gene repertoires in the analyzed vertebrate lineages. The red seven-pointed star indicates the local gene duplication that gave rise to ancestral vertebrate V1R and V2R genes. Red arrows indicate the vertebrate WGDs: 1R and 2R at the base of vertebrates, the teleost 3R at 320 million years ago (MYA) (39), the American paddlefish 3R at 50 MYA (18) and the sterlet 3R which has been estimated at both 180 MYA (17) and 21.3 MYA (19). Bars by red arrows indicate the estimated time windows for these WGD events in the cited literature. Time estimates of 1R and 2R vary wildly and are often overestimated, reaching back beyond the protostome-deuterostome split just over 600 MYA (40). Nonetheless, a time window of 600−450 MYA is reasonable based on estimates of the vertebrate-tunicate split, at the earliest, and the split between bony fishes and cartilaginous fishes, at the latest (40). The uncertain divergence of cyclostomes relative to 1R and 2R is indicated by a dashed branch. Light gray genes indicate the cyclostome OTR/VTR genes, which could not be unambiguously assigned to a subtype. The chordate phylogeny is based on divergence time estimations from 40, and TimeTree.org (http://timetree.org). Footnotes: 1) “Lizards” is a paraphyletic group including Schlegel’s Japanese gecko (*Gekko japonicus*), common wall lizard (*Podarcis muralis*) and Carolina anole lizard. 2) Some teleost species lack duplicate *VTR2B* genes (see ), and no *VTR2B* could be found in the Northern pike (*Esox lucius*). 3) Some teleost species lack *VTR2C* genes. 4) Teleost *VTR2C* genes cluster with the cartilaginous fish *VTR2C2* local duplicate (see **Figure 1**), possibly due to a combination of truncated C-terminals in both lineages and phylogenetic artifacts.

**Figure 9:**
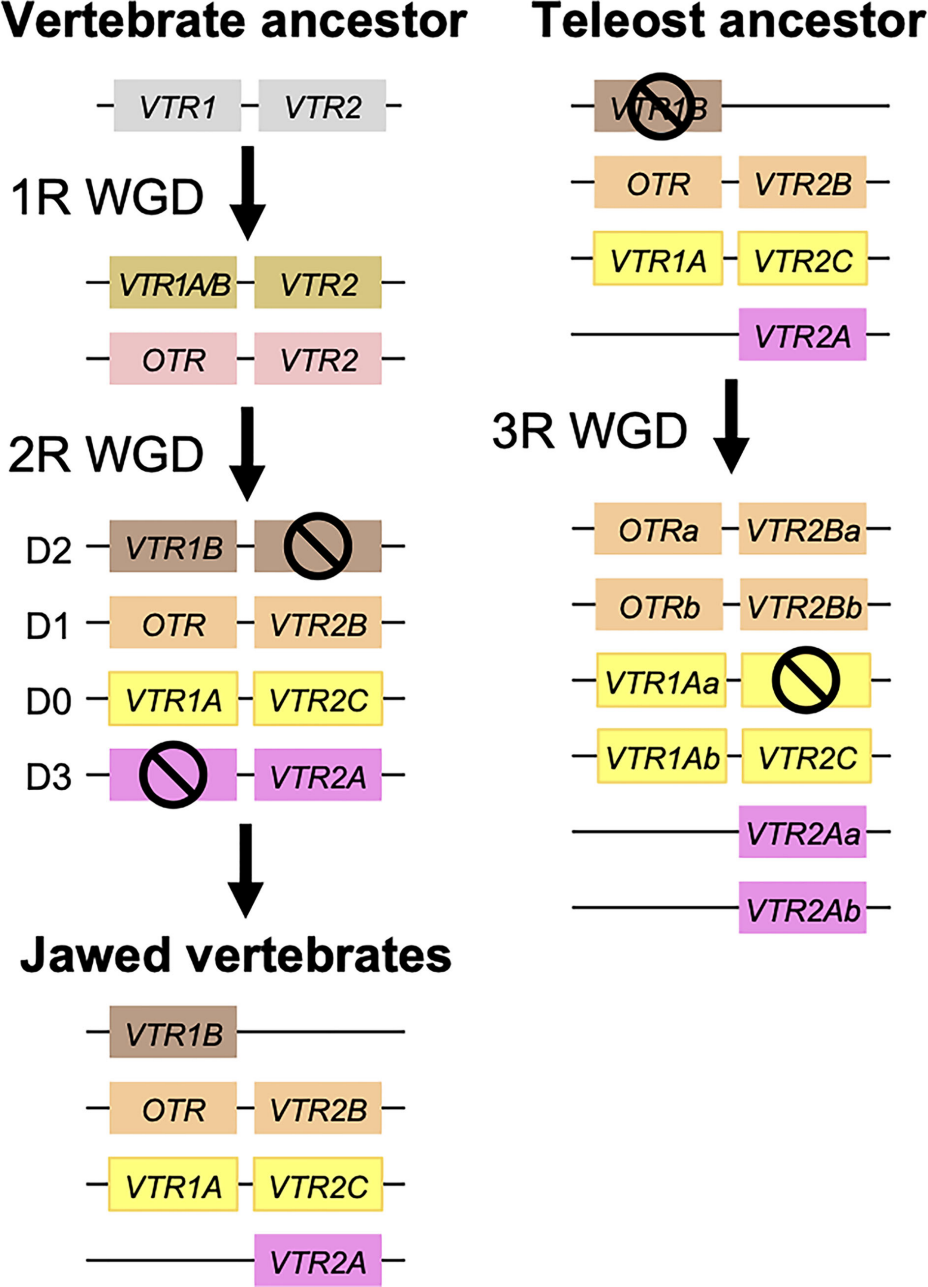
Duplication scheme of OTR/VTR genes through the 1R/2R and 3R whole-genome duplications. Gene losses following WGDs are indicated by ⦸ symbols. D0-D3 designations refer to paralogous chromosome blocks in jawed vertebrate genomes that originated in 1R/2R, as per (9).

Our suggested evolutionary scenario for the emergence and evolution of the OTR/VTR genes is shown in **Figure 8**, based on our updated phylogeny, species distribution and conserved synteny analyses. An ancestral OTR/VTR gene underwent a local gene duplication, giving rise to a VTR1/OTR ancestral gene and a VTR2 ancestral gene located on the same chromosome. This first local duplication took place a in chordate ancestor after the divergence of tunicates from the lineage leading to vertebrates, at the earliest, or in a vertebrate ancestor prior to the first of two WGD events, at the latest. Subsequently, these two ancestral genes duplicated in the 1R and 2R WGD events, as shown in **Figure 9**, after which one VTR1/OTR duplicate and one VTR2 duplicate were lost, leaving the ancestral jawed vertebrate subtype genes *VTR1A*, *VTR1B* and *OTR* on one side and *VTR2A*, *VTR2B* and *VTR2C* on the other. Due to the original local duplication, *VTR1A* and *VTR2C* as well as *OTR* and *VTR2B* remain located on the same chromosomes in many jawed vertebrate genomes.

Subsequently, there were several differential gene losses in several jawed vertebrate lineages. *VTR2A* was possibly lost from cartilaginous fishes, followed by the putative loss of *VTR1B* and *VTR2B* in sharks, rays and skates (Elasmobranchii) producing the smallest repertoire of OTR/VTR genes in any of the studied lineages. This is possibly related to the urea storage-based osmoregulatory system in cartilaginous fishes, which is unique among jawed vertebrates and whose endocrine control remains largely unknown (41). *VTR2A* also seems to have been lost from the lineage leading to Acipenseriformes, including the American paddlefish and sterlet, as well as from a common ancestor of birds and crocodilians. *VTR1B* was likely lost from the lineage leading to neopterygian fishes, including the holosteans spotted gar and bowfin as well as the teleosts. *VTR2B* was lost from the lineage leading to tetrapods after the divergence of the coelacanth lineage, while *VTR2C* was lost at least three times Sarcopterygii - from the coelacanth, mammals, and the lineage leading to caenophidian snakes.

The OTR/VTR genes duplicated further in the early teleost 3R WGD event, except for *VTR1B* which had been lost from neopterygian fishes (**Figure 9**). However, none of the analyzed teleost species preserve duplicate *VTR2C* genes. Subsequently *VTR2C* seems to have been lost independently several times from different teleost lineages. Likewise, several of the analyzed teleost species have lost either one or the other of the two *VTR2B* duplicates. We could also detect additional duplicates of *VTR1Aa*, *OTRa*, *OTRb*, *VTR2Aa*, *VTR2Ba* and *VTR2Bb* in the brown trout (*Salmo trutta*) genome (see **Supplementary Material**) that likely arose in the salmonid-specific 4R WGD event (42). The OTR/VTR genes also duplicated in the independent 3R WGD events in the lineages leading to the American paddlefish and the sterlet, except for *VTR2A* which likely had been lost in a common ancestor. The American paddlefish 3R has been dated at 50 MYA (18) and the sterlet 3R has been estimated at both 180 MYA (17) and 21.3 MYA (19).

### 3.3 Four-Fold Conserved Synteny in the OTR/VTR Chromosome Regions: A Clear Case for Gene Duplications Through Ancestral Whole-Genome Duplications

In a previously published study (12), we identified 34 neighboring gene families that showed patterns of conserved synteny in the chromosomal regions harboring OTR/VTR genes. For most of these neighboring gene families, 23 out of 34, phylogenetic analyses using two different methods (neighbor-joining and maximum likelihood) supported gene duplications in the time window of the 1R and 2R WGD events before the jawed vertebrate radiation. For the remaining 11 neighboring gene families identified in our previous study (12), a subset supported duplications in the time window of 1R/2R in one of the two phylogenetic methods, and yet another subset is compatible with duplications in 1R/2R but lacked an appropriate outgroup to relatively date the time window of duplications. Thus, we concluded that the chromosomal regions harboring these genes likely arose through 1R and 2R as well. This has been the gold standard for detecting the patterns of four-fold paralogy that resulted from 1R and 2R for nearly two decades, and we have been able to elucidate the evolution of many jawed vertebrate gene families through these ancient WGDs in this way. Notably, not all gene families that expanded prior to the jawed vertebrate radiation show signs of having duplicated through 1R and 2R. Out of the four jawed vertebrate genes that encode the growth hormone and prolactin family of hormones - *GH, SL, PRL1* and *PRL2* - only *PRL1* and *PRL2* arose through either of the 1R or 2R WGDs (43).

In the present study, we re-analyzed seven representative neighboring gene families (**Table 1**) out of the original 34 in the human, chicken, spotted gar and zebrafish genomes and added novel chromosomal data from the reedfish, thorny skate and Western clawed frog genomes. These seven gene families were chosen based on three criteria: 1) their clear and well-supported phylogenies in our previous study; 2) the presence of member genes on all four paralogous chromosome regions that were identified; and 3) their representation across the length of these paralogous regions. With these seven neighboring gene families, we could detect the same pattern of four-fold conserved synteny around the OTR/VTR genes in the Western clawed frog, reedfish and thorny skate genomes (**Figures 5**, **6**). This provides us with several key relative time points for the four-fold paralogy: at the divergence between cartilaginous fishes and bony fishes, relatively soon after the WGDs, at the base of ray−finned fishes, close to the divergence between ray-finned and lobe-finned fishes, and at the base of the tetrapod radiation. We could not identify the *VTR2A*-bearing chromosome region in the thorny skate genome (**Figure 5**). A possible explanation for this is that the thorny skate genome assembly provided by the Vertebrate Genomes Project was found to have a very high repeat content (54 %), which resulted in a large number of assembly gaps, even after manual modifications to the assembly pipeline and curation of the assembly to increase its quality (36). It is possible that the *VTR2A*-bearing chromosome region, and the *VTR2A* gene, are missing from the thorny skate genome assembly for this reason. The analyses of the white shark (*Carcharodon carcharias*), whale shark (*Rhincodon typus*), brownbanded bambooshark (*Chiloscyllium punctatum*) and cloudy catshark (*Scyliorhinus torazame*) genomes show a similarly high repeat content of approximately 50 % of the genome assemblies (33, 34). The repeat content of the elephant shark genome is also relatively high, at approximately 42 % [see Supplementary Note 4 in (33)]. We could not identify the *VTR2A* gene from any of the available cartilaginous fish genomes, and it is possible that this reflects the difficulty of integrating its surrounding chromosomal region into high-quality genome assemblies due to the presence of repeat sequences.

There have been several attempts at reconstructing the ancestral genome prior to the early vertebrate WGDs and to map the twice-duplicated ancestral chromosomes onto the genomes of extant vertebrate species. In the reconstruction by Nakatani et al. (9), the paralogous chromosome regions we have detected closely match the D0-D3 paralogous regions (marked in **Figure 9**) that arose through the quadruplication of ancestral linkage group D [see Figure 4 in (9)]. In the reconstruction by Putnam et al. (10), our paralogous chromosome regions match the four paralogous chromosomes that arose from the quadruplication of the ancestral chordate linkage group 13 [see Figure 3 in (10)]. The more recent reconstructions by Sacerdot et al. (44) and Simakov et al. (45) are also compatible with our findings. In the reconstruction by Sacerdot et al. (44), the paralogous regions we have detected closely match the reconstructed pre-1R chromosome 4 [see Figures 5 and 6 in (44)], and in the reconstruction by Simakov et al. (45) our paralogous regions in the human, chicken, Western clawed frog and spotted gar closely match the reconstructed chordate linkage group E [see Figure 2 in (45)]. The same pattern of four-fold paralogy has thus been detected by four independent studies, using different reconstruction methods, in addition to ours. Furthermore, the paralogous chromosome regions in teleost genomes, although heavily rearranged, match chromosome regions that have been shown to have originated in the teleost 3R WGD event in several independent studies (31, 46, 47).

### 3.4 Functional Implications

An important corollary of the whole-genome duplication scenario is that the gene duplicates initially most likely had not only identical coding sequences, but also identical regulatory regions. Thus, the *OTR, VTR1A* and *VTR1B* genes started as “identical triplets”, and likewise for the three *VTR2* genes. Subsequently, the *OTR*, *VTR1A* and *VTR1B* genes diverged functionally from each other resulting in three receptors with unique roles for each one, either by sub-functionalization (dividing the mother gene’s functions between the three daughter genes) or neo-functionalization (acquisition of novel functions). This would explain why they have been retained in the genomes of many vertebrate lineages (**Figure 8**). By the same token, the conservation of the three *VTR2* genes in diverse vertebrate lineages (**Figure 8**) suggests that they too have evolved unique functional roles. However, little has been done to investigate the functions of V2B and V2C receptors. Yamaguchi et al. (48) characterized some functional aspects of elephant shark and teleost V2B and V2C receptors and discovered that they elicit an intracellular Ca^2+^ cascade on activation, like V1A, V1B and OTR and unlike V2A, which engage the adenylyl cyclase/cAMP signal transduction pathway. It is also known that the chicken V2 receptor, which is a V2C, primarily signals *via* Ca2+ as a second messenger (49). Interestingly, the *VTR2B* gene appears to have been lost in a common ancestor of tetrapods, suggesting that its role became dispensable in this lineage, possibly in relation to the transition from water to land. It will be interesting to identify its unique role among other vertebrates. Furthermore, there seems to have been an asymmetric loss of V2C from a mammalian ancestor and V2A from an archosaurian ancestor of birds and crocodilians (**Figure 8**). This might be related to the fact that the kidneys in birds and crocodilians do not contribute significantly to water reabsorption. They rely instead on the intestine and well-developed salt glands for water/electrolyte balance. In domestic chickens the V2C receptor is primarily expressed in the shell gland and the brain (49). It will likewise be interesting to identify the unique role of *VTR2C* among other vertebrates, especially those that have both *VTR2A* and *VTR2C* genes, like turtles (Testudines).

Similarly, it will be interesting to find out how *VTR1B* could be lost in a neopterygian ancestor of bowfin, gars and teleosts but retained in earlier-diverging ray-finned fish lineages, like bichirs and reedfish as well as sturgeon.

Our analyses of the expanded IL3 regions of teleost V2A receptors also have functional implications. We found that these IL3 regions contain several relatively well-conserved Tyr, Ser and Thr residues, as well as a high degree of intrinsic disorder. These sequence features indicate a degree of functionality as well as similarities to sequence features seen in IL3 regions from other GPCRs. It has been shown that GPCRs can be phosphorylated on intracellular Tyr, Ser and Thr residues by various receptor kinases, mediating the modulation of intracellular signaling cascades as well as GPCR attenuation/desensitization, endocytosis and intracellular trafficking (50). The low functional activity of the relatively short IL3 of the human V2A receptor has been demonstrated through mutagenesis studies (51, 52), however for many GPCRs IL3 contains important sites for downstream interactions with G proteins, receptor kinases and arrestins, for example. It has also been shown that the IL3 regions of GPCRs in particular harbor intrinsically disordered stretches that facilitate such interactions (53–55), as well as GPCR oligomerization (56).

### 3.5 Do Recent Studies Refute the 1R/2R Scenario of OTR/VTR Evolution?

Recent studies of the evolution of OTR/VTR genes that included cyclostome sequences have questioned the double WGD evolutionary scenario and have proposed other schemes. One study concluded that the analyses supported one WGD event that was followed by “chromosomal or segmental duplications and translocations” in the jawed vertebrate lineage (13). This conclusion was repeated more recently in a book chapter from the same group (14). However, their analyses did not include neighboring gene families. Besides, it is well known that lamprey sequences tend to cluster in ambiguous positions in vertebrate phylogenies, partly due to a unique pattern of sequence evolution at the protein level as a result of GC-rich codons, but also possibly due to asymmetric gene retention after the WGD events between cyclostomes and jawed vertebrates (57, 58). It is also unclear when jawless vertebrates diverged from the branch leading to jawed vertebrates with respect to the two rounds of WGD (59). This makes early vertebrate branching events more difficult to interpret.

The most recent report on the evolution of OTR/VTR genes favored one WGD event followed by large segmental duplications in the jawed vertebrate lineage giving rise to *VTR1A* and *VTR1B* as well as *VTR2B* and *VTR2C* (4). This study included both lamprey and hagfish sequences. The analyses did include neighboring gene families, but comparisons were only performed between lamprey and various jawed vertebrates, not among the jawed vertebrates. Thus, the conclusion was most likely affected by the distant relationship to the cyclostomes, which makes conserved synteny difficult to interpret. We were able to identify the same sea lamprey and inshore hagfish sequences as the previously published study by Theofanopoulou et al. (4), and included them in our full phylogeny. However, based on the known set of problems when trying to infer the orthology between cyclostome and jawed vertebrate sequences, we don’t consider these results conclusive, and much caution should be taken before trying to reconcile the ambiguous positions of the cyclostome sequences within phylogenies with any scenario of OTR/VTR gene evolution. Furthermore, recent reports from ancestral genome reconstructions have suggested that cyclostomes share only the first WGD event (1R) with jawed vertebrates (27, 45) whereupon the cyclostome lineage underwent a genome triplication (hexaploidization) (27), further accentuating the complications related to comparisons between cyclostomes and jawed vertebrates. The study by Nakatani et al. from 2021 (27), nevertheless firmly concluded that the lineage leading to jawed vertebrates has undergone two WGD events. Aside from asymmetric gene duplicate retention after 1R, it is possible that further OTR/VTR gene duplicates could have arisen through different mechanisms in the two lineages: the second WGD in jawed vertebrates and hexaploidization in cyclostomes. We are currently carrying out further careful study of the chromosome regions in the most recent sea lamprey and inshore hagfish genome assemblies with the aim to resolve the evolutionary history of the cyclostome OTR/VTR genes.

Another reason why Theofanopoulou et al. (4) proposed that jawed vertebrates may have undergone only one WGD followed by several segmental duplications was probably that they could identify five, rather than four, chromosome segments showing conserved synteny [see Figure 2 in (4)]. However, their two blocks of genes on chromosome 3 are actually one and the same. Only three gene families are present in both blocks, and all three were analyzed incorrectly: 1) The *ARF* and *ARL8* genes do not form a quartet that arose in vertebrates, in fact *ARF* and *ARL8* existed much earlier in evolution; 2) The *ATP2B* genes form a quartet on the four human chromosomes, 1, 3, 12 and X (**Figure 5**), however the authors have omitted the *ATP2B1* gene on chromosome 12, misplaced the *ATP2B3* gene on chromosome 3 when it is in fact located on chromosome 12, and included *ATP6AP1*, which is not part of this gene family; and 3) the *CNTN3*, *-4*, and *-6* genes arose through a local gene triplication after 2R and the large distance to the *CNTN3* gene is due to a recent rearrangement in the primate lineage. Thus, all analyzed gene families in the study by Theofanopoulou et al. (4) nicely support chromosomal quadruplication, hence two WGD events.

Yet another reason that Theofanopoulou et al. (4) proposed segmental duplications in jawed vertebrates is the lack of one of the four paralogous chromosome regions (bearing *VTR2A*) in the elephant shark genome, indicating that it might have arisen in a bony fish (Osteichthyes) ancestor. Our extended analysis of seven additional species confirms that this chromosome region indeed seems to be missing from cartilaginous fishes. However, as we discuss above, we cannot reject the possibility that these regions are missing from cartilaginous fish genome assemblies due to their high repeat content. We could not identify the *VTR2A*-bearing chromosome region in avian genomes either. Large chromosomal regions can indeed be lost, as has been shown for *HOX* clusters in teleosts after the 3R event.

Our sequence-based phylogenies as well as conserved synteny analysis strongly support an origin of all four paralogous chromosome regions before the jawed vertebrate radiation. The distinct four-fold paralogy is preserved in diverse jawed vertebrate lineages and consists of gene families that diverged early in vertebrate evolution. This provides a clear case for gene duplication through the 1R and 2R WGD events.

### 3.6 OTR/VTR Gene Nomenclature

We previously suggested a simplified nomenclature for the family of six OT/VT receptors in vertebrates, V1A, V1B, OTR, V2A, V2B and V2C, based on both phylogenetic analysis and chromosomal location (11, 12). This nomenclature has so far been used in a number of publications (13, 48, 60–64). Recently, Theofanopoulou et al. (4), suggested naming the OTR/VTR genes *VTR1A, VTR1B, OTR, VTR2A, VTR2B* and *VTR2C*, foregoing the letter A for *arginine*-vasopressin currently used in the accepted gene names. We support this proposal and have used this nomenclature throughout the present study. However, Theofanopoulou et al. (4) have interchanged the *VTR2A* and *VTR2C* designations based on an inferred chronological order of emergence of the genes, suggesting that *VTR2A* (which they call *VTR2C*) and its chromosomal region arose later, in a bony fish ancestor. They base this order on the species distribution of *VTR2A* genes and their attempt to assign the lamprey V2R-type genes to specific subtypes. However, as we have shown, the *VTR2A, VTR2B* and *VTR2C* genes arose concomitantly in the 1R/2R WGD events, not in sequence, and the lamprey V2R-type genes have not yet been confidently assigned to specific subtypes. For these reasons we strongly suggest retaining the original ABC designations as they a) have already been used consistently in the literature for nearly a decade; and b) the genes arose concomitantly in the 1R/2R WGD events, not in a chronological sequence, as Theofanopoulou et al. (4) propose.

## 4 Materials and Methods

### 4.1 Identification of OTR/VTR Genes

The OTR/VTR gene models/sequences identified in our previous study (12) were updated, where applicable, to the corresponding data from the latest available genome assemblies in the National Center for Biotechnology Information (NCBI) Genome database (http://www.ncbi.nlm.nih.gov/genome/) (65). The corresponding chromosomal location data were also updated. OTR/VTR gene models/sequences from additional vertebrate species were identified in the NCBI database using the same BLAST-based method as in our previous study (12), and added to the dataset. All identified OTR/VTR gene models/sequences, with their corresponding location data and database identifiers are available in Figshare (see **Supplementary Material**). Species, genome assembly and database information are shown in **Table 1**.

### 4.2 Sequence Alignment and Phylogenetic Analysis

The identified OTR/VTR amino acid sequences were aligned using the MUSCLE algorithm (66) in AliView version 1.6 (67). The alignment ﻿was edited manually to adjust poorly aligned sequence stretches with respect to conserved motifs, exon boundaries and incomplete sequences. Missing sequence stretches caused by gaps in the genome assemblies were replaced with X-positions. Partial sequences are marked with asterisks (*) in the final alignment. The curated alignment was used to create a maximum-likelihood phylogeny using IQ-TREE (68) version 1.6.12 (August 15, 2019) supported by IQ-TREE’s non-parametric UltraFast Bootstrap (UFBoot) method (100 iterations) (69). The best-fit amino acid substitution model and substitution parameters were estimated using IQ-TREE’s ModelFinder with the -m TEST option (70), and the proportion of invariant sites was optimized by using the –opt−gamma−inv option. The phylogeny was rooted with the common octopus (*Octopus vulgaris*) cephalotocin and octopressin receptor sequences (71).

### 4.3 Conserved Synteny Analyses

The following neighboring gene families from (12), are included: ATP2B (ATPase plasma membrane Ca^2+^ transporting protein B), CACNA1-L (Voltage-gated Ca^2+^ channel alpha-1 subunit, L-type), CAMK1 (Calcium/calmodulin dependent protein kinase 1 family), L1CAM (L1-related cell adhesion molecules), PLXNA (Plexin A family), SRGAP (SLIT-ROBO Rho GTPase activating protein), and SYP (Synaptophysin/synaptoporin). Briefly, these and other neighboring gene families were identified by comparing the gene composition of the chromosomal regions bearing visual opsin, OTR/VTR, *CACNA1-L*, *GNAT* and *GNAI* genes in the human genome. Those gene families with member genes on at least two chromosomal regions were selected for further analysis [see Methods in (12)].

Gene models and locations for all neighboring genes were updated to the corresponding data from the latest available genome assemblies in the NCBI Genome database. In addition, neighboring family gene model and location data from the Western clawed frog, reedfish and thorny skate were added in order to identify the paralogous OTR/VTR-bearing chromosome regions in these key species.

For the conserved synteny analysis of 3R-generated paralogous regions in the Asian arowana (*Sleropages formosus*), the conserved neighboring gene pairs were identified through Genomicus v100.01 (72) and the time window of gene pair emergence was inferred from the corresponding Ensembl gene trees (73). Where the orthology relationships to the zebrafish genes was clear in the Ensembl gene trees, gene names with “a” and “b”-designations have been suggested. Where there was only one zebrafish ortholog, this was designated the “a”-duplicate.

## Data Availability Statement

The datasets generated and analyzed for this study have been deposited in Figshare (https://www.figshare.com): https://doi.org/10.6084/m9.figshare.16125318 (see also **Supplementary Material**).

## Author Contributions

DO and CB collected the data and performed primary analyses. DO and DL analyzed the data and wrote the manuscript. All authors contributed to the article and approved the submitted version.

## Funding

DO was supported by International Postdoc Grant 2016-00552 from the Swedish Research Council. DL was supported by grants from the Swedish Brain Foundation and the Swedish Research Council.

## Conflict of Interest

The authors declare that the research was conducted in the absence of any commercial or financial relationships that could be construed as a potential conflict of interest.

## Publisher’s Note

All claims expressed in this article are solely those of the authors and do not necessarily represent those of their affiliated organizations, or those of the publisher, the editors and the reviewers. Any product that may be evaluated in this article, or claim that may be made by its manufacturer, is not guaranteed or endorsed by the publisher.

## Supplementary Material

The datasets generated and analyzed for this study are available in Figshare via https://doi.org/10.6084/m9.figshare.16125318.

Sequence and sequence alignment files are included in FASTA format, which can be opened by most sequence alignment viewers, including online applications such as https://alignmentviewer.org/ (last accessed 2021-09-27). An annotated rich text file (file name extension.rtf) document which shows the exon/intron boundaries for all analyzed sequences has also been included. Phylogenetic tree files are included in the NEWICK format (file name extension .phb), which can be opened by most phylogenetic tree viewers, including online applications such as https://itol.embl.de/, http://etetoolkit.org/treeview/ or https://icytree.org/ (last accessed 2021-09-27). The raw output file packages from IQ-TREE have also been included.

## References

[1] Pittman QJ. Vasopressin and Central Control of the Cardiovascular System: A 40-Year Retrospective. J Neuroendocrinol (2021) 33(11):1–6. doi: 10.1111/jne.13011 34235812

[2] Acher R. Molecular Evolution of Fish Neurohypophysial Hormones: Neutral and Selective Evolutionary Mechanisms. Gen Comp Endocrinol (1996) 102:157–72. doi: 10.1006/gcen.1996.0057 8998960

[3] Gwee P-C Tay B-H Brenner S Venkatesh B. Characterization of the Neurohypophysial Hormone Gene Loci in Elephant Shark and the Japanese Lamprey: Origin of the Vertebrate Neurohypophysial Hormone Genes. BMC Evol Biol (2009) 9(47):1–15. doi: 10.1186/1471-2148-9-47 19243634 PMC2656470

[4] Theofanopoulou C Gedman G Cahill JA Boeckx C Jarvis ED. Universal Nomenclature for Oxytocin–Vasotocin Ligand and Receptor Families. Nature (2021) 592:747–55. doi: 10.1038/s41586-020-03040-7 PMC8081664 33911268

[5] Elphick MR Mirabeau O Larhammar D. Evolution of Neuropeptide Signalling Systems. J Exp Biol (2018) 221(2):1–15. doi: 10.1242/jeb.151092 PMC5818035 29440283

[6] Iovino M Messana T Tortora A Giusti C Lisco G Giagulli VA. Oxytocin Signaling Pathway: From Cell Biology to Clinical Implications. Endocr Metab Immune Disord Drug Targets (2021) 21:91–110. doi: 10.2174/1871530320666200520093730 32433011

[7] Birnbaumer M Seibold A Gilbert S Ishido M Barberis C Antaramian A. Molecular Cloning of the Receptor for Human Antidiuretic Hormone. Nature (1992) 357:333–5. doi: 10.1038/357333a0 1534149

[8] Zeynalov E Jones SM Elliott JP. Vasopressin and Vasopressin Receptors in Brain Edema. Vitam Horm (2020) 113:291–312. doi: 10.1016/bs.vh.2019.08.015 32138953

[9] Nakatani Y Takeda H Kohara Y Morishita S. Reconstruction of the Vertebrate Ancestral Genome Reveals Dynamic Genome Reorganization in Early Vertebrates. Genome Res (2007) 17:1254–65. doi: 10.1101/gr.6316407 PMC1950894 17652425

[10] Putnam NH Butts T Ferrier DEK Furlong RF Hellsten U Kawashima T. The Amphioxus Genome and the Evolution of the Chordate Karyotype. Nature (2008) 453:1064–71. doi: 10.1038/nature06967 18563158

[11] Ocampo Daza D Lewicka M Larhammar D. The Oxytocin/Vasopressin Receptor Family has at Least Five Members in the Gnathostome Lineage, Including Two Distinct V2 Subtypes. Gen Comp Endocrinol (2012) 175:135–43. doi: 10.1016/j.ygcen.2011.10.011 22057000

[12] Lagman D Ocampo Daza D Widmark J Abalo XM Sundström G Larhammar D. The Vertebrate Ancestral Repertoire of Visual Opsins, Transducin Alpha Subunits and Oxytocin/Vasopressin Receptors was Established by Duplication of Their Shared Genomic Region in the Two Rounds of Early Vertebrate Genome Duplications. BMC Evol Biol (2013) 13:238. doi: 10.1186/1471-2148-13-238 24180662 PMC3826523

[13] Mayasich SA Clarke BL. The Emergence of the Vasopressin and Oxytocin Hormone Receptor Gene Family Lineage: Clues From the Characterization of Vasotocin Receptors in the Sea Lamprey (Petromyzon Marinus). Gen Comp Endocrinol (2016) 226:88–101. doi: 10.1016/j.ygcen.2016.01.001 26764211

[14] Mayasich SA Clarke BL. “Vasotocin and the Origins of the Vasopressin/Oxytocin Receptor Gene Family”. In: Vitamins and Hormones. Vol. 113: Vasopressin Elsevier Inc (2020). p. 1–27. doi: 10.1016/bs.vh.2019.08.018 32138945

[15] Ocampo Daza D Lewicka M Venkatesh B Larhammar D. The Oxytocin/Vasopressin Receptor Family has at Least Five Members in the Gnathostome Lineage. Front Endocrinol (Lausanne) (2011) 2:2011.04.00083. doi: 10.3389/conf.fendo.2011.04.00083

[16] Gilbert SF Corfe I. Turtle Origins: Picking Up Speed. Dev Cell (2013) 25:326–8. doi: 10.1016/j.devcel.2013.05.011 23725759

[17] Du K Stöck M Kneitz S Klopp C Woltering JM Adolfi MC. The Sterlet Sturgeon Genome Sequence and the Mechanisms of Segmental Rediploidization. Nat Ecol Evol (2020) 4:841–52. doi: 10.1038/s41559-020-1166-x PMC7269910 32231327

[18] Cheng P Huang Y Lv Y Du H Ruan Z Li C. The American Paddlefish Genome Provides Novel Insights Into Chromosomal Evolution and Bone Mineralization in Early Vertebrates. Mol Biol Evol (2021) 38:1595–607. doi: 10.1093/molbev/msaa326 PMC8042750 33331879

[19] Cheng P Huang Y Du H Li C Lv Y Ruan R. Draft Genome and Complete Hox-Cluster Characterization of the Sterlet Sturgeon (Acipenser Ruthenus). Front Genet (2019) 10:776. doi: 10.3389/fgene.2019.00776 31543900 PMC6739705

[20] Onimaru K Tatsumi K Shibagaki K Kuraku S. A *De Novo* Transcriptome Assembly of the Zebra Bullhead Shark, Heterodontus Zebra. Sci Data (2018) 5:180197. doi: 10.1038/sdata.2018.197 30295671 PMC6174923

[21] Tanegashima C Nishimura O Motone F Tatsumi K Kadota M Kuraku S. Embryonic Transcriptome Sequencing of the Ocellate Spot Skate Okamejei Kenojei. Sci Data (2018) 5:180200. doi: 10.1038/sdata.2018.200 30295675 PMC6174922

[22] Machado AM Almeida T Mucientes G Esteves PJ Verissimo A Castro LFC. *De Novo* Assembly of the Kidney and Spleen Transcriptomes of the Cosmopolitan Blue Shark, Prionace Glauca. Mar Genomics (2018) 37:50–3. doi: 10.1016/j.margen.2017.11.009 33250128

[23] Redmond AK Macqueen DJ Dooley H. Phylotranscriptomics Suggests the Jawed Vertebrate Ancestor Could Generate Diverse Helper and Regulatory T Cell Subsets. BMC Evol Biol (2018) 18:1–19. doi: 10.1186/s12862-018-1290-2 30442091 PMC6238376

[24] Lighten J Incarnato D Ward BJ van Oosterhout C Bradbury I Hanson M. Adaptive Phenotypic Response to Climate Enabled by Epigenetics in a K-Strategy Species, the Fish Leucoraja Ocellata (Rajidae). R Soc Open Sci (2016) 3:160299. doi: 10.1098/rsos.160299 27853546 PMC5098971

[25] Mulley JF Hargreaves AD Hegarty MJ Heller RS Swain MT. Transcriptomic Analysis of the Lesser Spotted Catshark (Scyliorhinus Canicula) Pancreas, Liver and Brain Reveals Molecular Level Conservation of Vertebrate Pancreas Function. BMC Genomics (2014) 15:1–18. doi: 10.1186/1471-2164-15-1074 25480530 PMC4362833

[26] Vidal N Delmas AS David P Cruaud C Couloux A Hedges SB. The Phylogeny and Classification of Caenophidian Snakes Inferred From Seven Nuclear Protein-Coding Genes. Comptes Rendus - Biol (2007) 330:182–7. doi: 10.1016/j.crvi.2006.10.001 17303545

[27] Nakatani Y Shingate P Ravi V Pillai NE Prasad A McLysaght A. Reconstruction of Proto-Vertebrate, Proto-Cyclostome and Proto-Gnathostome Genomes Provides New Insights Into Early Vertebrate Evolution. Nat Commun (2021) 12:1–14. doi: 10.1038/s41467-021-24573-z 34301952 PMC8302630

[28] Böselt I Römpler H Hermsdorf T Thor D Busch W Schulz A. Involvement of the V2 Vasopressin Receptor in Adaptation to Limited Water Supply. PloS One (2009) 4:e5573. doi: 10.1371/journal.pone.0005573 19440390 PMC2680020

[29] Mészáros B Erdös G Dosztányi Z. IUPred2A: Context-Dependent Prediction of Protein Disorder as a Function of Redox State and Protein Binding. Nucleic Acids Res (2018) 46:W329–37. doi: 10.1093/nar/gky384 PMC6030935 29860432

[30] Erdős G Dosztányi Z. Analyzing Protein Disorder With IUPred2A. Curr Protoc Bioinform (2020) 70:1–15. doi: 10.1002/cpbi.99 32237272

[31] Nakatani Y McLysaght A. Genomes as Documents of Evolutionary History: A Probabilistic Macrosynteny Model for the Reconstruction of Ancestral Genomes. Bioinformatics (2017) 33:i369–78. doi: 10.1093/bioinformatics/btx259 PMC5870716 28881993

[32] Read TD Petit RA Joseph SJ Alam MT Weil MR Ahmad M. Draft Sequencing and Assembly of the Genome of the World’s Largest Fish, the Whale Shark: Rhincodon Typus Smith 1828. BMC Genomics (2017) 18:1–10. doi: 10.1186/s12864-017-3926-9 28709399 PMC5513125

[33] Hara Y Yamaguchi K Onimaru K Kadota M Koyanagi M Keeley SD. Shark Genomes Provide Insights Into Elasmobranch Evolution and the Origin of Vertebrates. Nat Ecol Evol (2018) 2:1761–71. doi: 10.1038/s41559-018-0673-5 30297745

[34] Marra NJ Stanhope MJ Jue NK Wang M Sun Q Pavinski Bitar P. White Shark Genome Reveals Ancient Elasmobranch Adaptations Associated With Wound Healing and the Maintenance of Genome Stability. Proc Natl Acad Sci (2019) 116(10):4446–55. doi: 10.1073/pnas.1819778116 PMC6410855 30782839

[35] Zhang Y Gao H Li H Guo J Ouyang B Wang M. The White-Spotted Bamboo Shark Genome Reveals Chromosome Rearrangements and Fast-Evolving Immune Genes of Cartilaginous Fish. iScience (2020) 23:101754. doi: 10.1016/j.isci.2020.101754 33251490 PMC7677710

[36] Rhie A McCarthy SA Fedrigo O Damas J Formenti G Koren S. Towards Complete and Error-Free Genome Assemblies of All Vertebrate Species. Nature (2021) 592:737–46. doi: 10.1038/s41586-021-03451-0 PMC8081667 33911273

[37] Yamaguchi K Hara Y Tatsumi K Nishimura O Jeramiah J. Inference of a Genome-Wide Protein-Coding Gene Set of the Inshore Hagfish Eptatretus Burgeri. bioRxiv (2020). doi: 10.1101/2020.07.24.218818

[38] Hess JE Smith JJ Timoshevskaya N Baker C Caudill CC Graves D. Genomic Islands of Divergence Infer a Phenotypic Landscape in Pacific Lamprey. Mol Ecol (2020) 29:3841–56. doi: 10.1111/mec.15605 32814354

[39] Vandepoele K De Vos W Taylor JS Meyer A van de Peer Y. Major Events in the Genome Evolution of Vertebrates: Paranome Age and Size Differ Considerably Between Ray-Finned Fishes and Land Vertebrates. Proc Natl Acad Sci USA (2004) 101:1638–43. doi: 10.1073/pnas.0307968100 PMC341801 14757817

[40] dos Reis M Thawornwattana Y Angelis K Telford MJ Donoghue PCJ Yang Z. Uncertainty in the Timing of Origin of Animals and the Limits of Precision in Molecular Timescales. Curr Biol (2015) 25:2939–50. doi: 10.1016/j.cub.2015.09.066 PMC4651906 26603774

[41] Hazon N Wells A Pillans RD Good JP Anderson WG Franklin CE. Urea Based Osmoregulation and Endocrine Control in Elasmobranch Fish With Special Reference to Euryhalinity. Comp Biochem Physiol - B Biochem Mol Biol (2003) 136:685–700. doi: 10.1016/S1096-4959(03)00280-X 14662294

[42] Glasauer SMK Neuhauss SCF. Whole-Genome Duplication in Teleost Fishes and its Evolutionary Consequences. Mol Genet Genomics (2014) 289:1045–60. doi: 10.1007/s00438-014-0889-2 25092473

[43] Ocampo Daza D Larhammar D. Evolution of the Growth Hormone, Prolactin, Prolactin 2 and Somatolactin Family. Gen Comp Endocrinol (2018) 264:94–112. doi: 10.1016/j.ygcen.2018.01.007 29339183

[44] Sacerdot C Louis A Bon C Berthelot C Roest Crollius H. Chromosome Evolution at the Origin of the Ancestral Vertebrate Genome. Genome Biol (2018) 19:166. doi: 10.1186/s13059-018-1559-1 30333059 PMC6193309

[45] Simakov O Marlétaz F Yue JX O’Connell B Jenkins J Brandt A. Deeply Conserved Synteny Resolves Early Events in Vertebrate Evolution. Nat Ecol Evol (2020) 4:820–30. doi: 10.1038/s41559-020-1156-z PMC7269912 32313176

[46] Kasahara M Naruse K Sasaki S Nakatani Y Qu W Ahsan B. The Medaka Draft Genome and Insights Into Vertebrate Genome Evolution. Nature (2007) 447:714–9. doi: 10.1038/nature05846 17554307

[47] Bian C Hu Y Ravi V Kuznetsova IS Shen X Mu X. The Asian Arowana (Scleropages Formosus) Genome Provides New Insights Into the Evolution of an Early Lineage of Teleosts. Sci Rep (2016) 6:24501. doi: 10.1038/srep24501 27089831 PMC4835728

[48] Yamaguchi Y Kaiya H Konno N Iwata E Miyazato M Uchiyama M. The Fifth Neurohypophysial Hormone Receptor is Structurally Related to the V2-Type Receptor But Functionally Similar to V1-Type Receptors. Gen Comp Endocrinol (2012) 178:519–28. doi: 10.1016/j.ygcen.2012.07.008 22809669

[49] Tan F Lolait SJ Brownstein MJ Saito N MacLeod V Baeyens DA. Molecular Cloning and Functional Characterization of a Vasotocin Receptor Subtype That is Expressed in the Shell Gland and Brain of the Domestic Chicken. Biol Reprod (2000) 62:8–15. doi: 10.1095/biolreprod62.1.8 10611061

[50] Tobin AB Butcher AJ Kong KC. Location, Location, Location...Site-Specific GPCR Phosphorylation Offers a Mechanism for Cell-Type-Specific Signalling. Trends Pharmacol Sci (2008) 29:413–20. doi: 10.1016/j.tips.2008.05.006 PMC2880250 18606460

[51] Pan Y Wilson P Gitschier J. The Effect of Eight V2 Vasopressin Receptor Mutations on Stimulation of Adenylyl Cyclase and Binding to Vasopressin. wfi 2J Biol Chem (1994) 269:31933–7. doi: 10.1016/S0021-9258(18)31785-X 7527400

[52] Schoneberg T Yun J Wenkert D Wess J. Functional Rescue of Mutant V2 Vasopressin Receptors Causing Nephrogenic Diabetes Insipidus by a Co-Expressed Receptor Polypeptide. EMBO J (1996) 15:1283–91. doi: 10.1002/j.1460-2075.1996.tb00470.x PMC450031 8635461

[53] Jaakola VP Prilusky J Sussman JL Goldman A. G Protein-Coupled Receptors Show Unusual Patterns of Intrinsic Unfolding. Protein Eng Des Sel (2005) 18:103–10. doi: 10.1093/protein/gzi004 15790574

[54] Agnati LF Leo G Genedani S Andreoli N Marcellino D Woods A. Structural Plasticity in G-Protein Coupled Receptors as Demonstrated by the Allosteric Actions of Homocysteine and Computer-Assisted Analysis of Disordered Domains. Brain Res Rev (2008) 58:459–74. doi: 10.1016/j.brainresrev.2007.10.003 18022243

[55] Venkatakrishnan AJ Flock T Prado DE Oates ME Gough J Madan Babu M. Structured and Disordered Facets of the GPCR Fold. Curr Opin Struct Biol (2014) 27:129–37. doi: 10.1016/j.sbi.2014.08.002 25198166

[56] Farran B. An Update on the Physiological and Therapeutic Relevance of GPCR Oligomers. Pharmacol Res (2017) 117:303–27. doi: 10.1016/j.phrs.2017.01.008 28087443

[57] Kuraku S. Palaeophylogenomics of the Vertebrate Ancestor–Impact of Hidden Paralogy on Hagfish and Lamprey Gene Phylogeny. Integr Comp Biol (2010) 50:124–9. doi: 10.1093/icb/icq044 21558193

[58] Kuraku S. Impact of Asymmetric Gene Repertoire Between Cyclostomes and Gnathostomes. Semin Cell Dev Biol (2013) 24:119–27. doi: 10.1016/j.semcdb.2012.12.009 23291292

[59] Kuraku S Meyer A Kuratani S. Timing of Genome Duplications Relative to the Origin of the Vertebrates: Did Cyclostomes Diverge Before or After? Mol Biol Evol (2009) 26:47–59. doi: 10.1093/molbev/msn222 18842688

[60] Hasunuma I Sakai T Nakada T Toyoda F Namiki H Kikuyama S. Molecular Cloning of Three Types of Arginine Vasotocin Receptor in the Newt, Cynops Pyrrhogaster. Gen Comp Endocrinol (2007) 151:252–8. doi: 10.1016/j.ygcen.2007.02.002 17367790

[61] Hasunuma I Toyoda F Okada R Yamamoto K Kadono Y Kikuyama S. *Roles of Arginine Vasotocin Receptors in the Brain and Pituitary of Submammalian Vertebrates. 1st ed.* Elsevier Inc. Int Rev Cell Mol Biol (2013) 304:191–225. doi: 10.1016/B978-0-12-407696-9.00004-X 23809437

[62] Kelly AM Goodson JL. Social Functions of Individual Vasopressin-Oxytocin Cell Groups in Vertebrates: What Do We Really Know? Front Neuroendocrinol (2014) 35(4):512–29. doi: 10.1016/j.yfrne.2014.04.005 24813923

[63] Lema SC Sanders KE Walti KA. Arginine Vasotocin, Isotocin and Nonapeptide Receptor Gene Expression Link to Social Status and Aggression in Sex-Dependent Patterns. J Neuroendocrinol (2015) 27:142–57. doi: 10.1111/jne.12239 25425529

[64] Rawat A Chaube R Joy KP. Molecular Cloning, Sequencing and Phylogeny of Vasotocin Receptor Genes in the Air-Breathing Catfish Heteropneustes Fossilis With Sex Dimorphic and Seasonal Variations in Tissue Expression. Fish Physiol Biochem (2015) 41:509–32. doi: 10.1007/s10695-015-0026-0 25596856

[65] Sayers EW Beck J Bolton EE Bourexis D Brister JR Canese K. Database Resources of the National Center for Biotechnology Information. Nucleic Acids Res (2021) 49:D10–7. doi: 10.1093/nar/gkaa892 PMC7778943 33095870

[66] Edgar RC. MUSCLE: Multiple Sequence Alignment With High Accuracy and High Throughput. Nucleic Acids Res (2004) 32:1792–7. doi: 10.1093/nar/gkh340 PMC390337 15034147

[67] Larsson A. AliView: A Fast and Lightweight Alignment Viewer and Editor for Large Data Sets. Bioinformatics (2014) 30:btu531. doi: 10.1093/bioinformatics/btu531 PMC4221126 25095880

[68] Nguyen LT Schmidt HA Von Haeseler A Minh BQ. IQ-TREE: A Fast and Effective Stochastic Algorithm for Estimating Maximum-Likelihood Phylogenies. Mol Biol Evol (2015) 32:268–74. doi: 10.1093/molbev/msu300 PMC4271533 25371430

[69] Minh BQ Nguyen MAT Von Haeseler A. Ultrafast Approximation for Phylogenetic Bootstrap. Mol Biol Evol (2013) 30:1188–95. doi: 10.1093/molbev/mst024 PMC3670741 23418397

[70] Kalyaanamoorthy S Minh BQ Wong TKF Von Haeseler A Jermiin LS. ModelFinder: Fast Model Selection for Accurate Phylogenetic Estimates. Nat Methods (2017) 14:587–9. doi: 10.1038/nmeth.4285 PMC5453245 28481363

[71] Kanda A Satake H Kawada T Minakata H. Novel Evolutionary Lineages of the Invertebrate Oxytocin/Vasopressin Superfamily Peptides and Their Receptors in the Common Octopus (Octopus Vulgaris). Biochem J (2005) 387:85–91. doi: 10.1042/BJ20041230 15504101 PMC1134935

[72] Nguyen NTT Vincens P Crollius HR Louis A. Genomicus 2018: Karyotype Evolutionary Trees and on-the-Fly Synteny Computing. Nucleic Acids Res (2018) 46:D816–22. doi: 10.1093/nar/gkx1003 PMC5753199 29087490

[73] Vilella AJ Severin J Ureta-Vidal A Heng L Durbin R Birney E. EnsemblCompara GeneTrees: Complete, Duplication-Aware Phylogenetic Trees in Vertebrates. Genome Res (2009) 19:327–35. doi: 10.1101/gr.073585.107 PMC2652215 19029536

